# Actinomycete-Derived Polyketides as a Source of Antibiotics and Lead Structures for the Development of New Antimicrobial Drugs

**DOI:** 10.3390/antibiotics8040157

**Published:** 2019-09-20

**Authors:** Helene L. Robertsen, Ewa M. Musiol-Kroll

**Affiliations:** Interfakultäres Institut für Mikrobiologie und Infektionsmedizin, Eberhard Karls Universität Tübingen, Auf der Morgenstelle 28, 72076 Tübingen, Germany; helene.robertsen@biotech.uni-tuebingen.de

**Keywords:** actinomycetes, bioactivity, antimicrobials, polyketides, polyketide synthases, biosynthesis

## Abstract

Actinomycetes are remarkable producers of compounds essential for human and veterinary medicine as well as for agriculture. The genomes of those microorganisms possess several sets of genes (biosynthetic gene cluster (BGC)) encoding pathways for the production of the valuable secondary metabolites. A significant proportion of the identified BGCs in actinomycetes encode pathways for the biosynthesis of polyketide compounds, nonribosomal peptides, or hybrid products resulting from the combination of both polyketide synthases (PKSs) and nonribosomal peptide synthetases (NRPSs). The potency of these molecules, in terms of bioactivity, was recognized in the 1940s, and started the “Golden Age” of antimicrobial drug discovery. Since then, several valuable polyketide drugs, such as erythromycin A, tylosin, monensin A, rifamycin, tetracyclines, amphotericin B, and many others were isolated from actinomycetes. This review covers the most relevant actinomycetes-derived polyketide drugs with antimicrobial activity, including anti-fungal agents. We provide an overview of the source of the compounds, structure of the molecules, the biosynthetic principle, bioactivity and mechanisms of action, and the current stage of development. This review emphasizes the importance of actinomycetes-derived antimicrobial polyketides and should serve as a “lexicon”, not only to scientists from the Natural Products field, but also to clinicians and others interested in this topic.

## 1. Introduction

**Figure 1:**
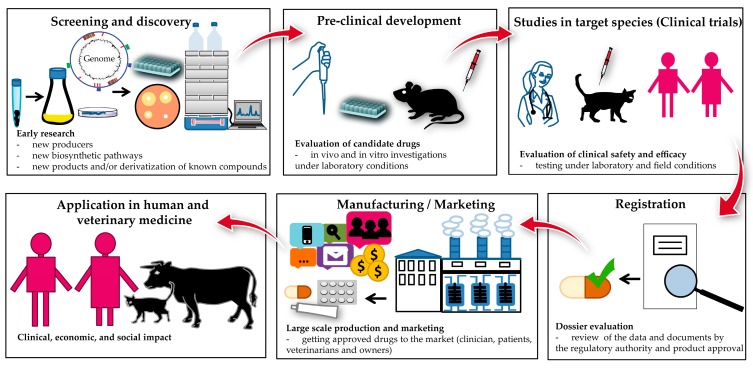
The process of drug development.

More than 70 years has passed since the discovery of the first antibiotics from actinomycetes. Although some of the high G+C (bacteria with high guanine- (G) and cytosine- (C) content in their genomes) [1,2], Gram-positive bacteria of the order *Actinomycetales* and their products have been studied in depth, they remain one of the most important sources of secondary metabolites, including the naturally-derived antimicrobial drugs (e.g., β-lactams, tetracyclines, rifamycins, macrolides, aminoglycosides, and glycopeptides). In the past, the producers of valuable bioactive compounds were mainly isolated from soil samples and either directly or indirectly, using culture filtrates or extracts, subjected to susceptibility testing (diffusion method) (Figure 1). Recent advances in different disciplines of science, such as robotics [3], biology [4], chemistry [5,6,7], genetics [8], and/or bioinformatics [9] has extended the spectrum of methodology applied to the isolation and identification of actinomycetes and the produced metabolites. In addition to technical innovations which, for example, enable sampling of unexplored and difficult to access environments, Next Generation Sequencing Technologies (NGST) and genome mining facilitate accurate and cost-effective sequencing of actinomycetes genomes and a fast identification of genes encoding the proteins of the secondary metabolite biosynthetic machineries (biosynthetic gene clusters, BGCs). In the past two decades, hundreds of actinomycetes genomes have been sequenced and many of them have been fully annotated. The analysis of the data has demonstrated that a significant portion of the BGCs are associated with polyketide synthase (PKS) and nonribosomal peptide synthetase (NRPS) pathways, indicating that polyketides, nonribosomal peptides, and their hybrid compounds are the major secondary metabolites of actinomycetes [10,11]. The biosynthesis of both, polyketides and nonribosomal peptides, involves multifunctional megaenzymes referred as PKSs and NRPSs, respectively (Section 2). Despite the fact that polyketides are assembled according to a similar biosynthetic principle, a wide variety of chemical structures are found in the producer strains. This structural diversity is mainly accomplished through the different architecture and specificity of the PKSs, variation of the building blocks for the polyketide chain biosynthesis, and post-PKS modification reaction. Consequently, it is expected that the chemical arsenal provided by polyketide pathways encompasses molecules targeting various cellular compartments or interacting with distinct sites of the same target. Indeed, polyketide drugs such as erythromycins and rifamycins have a different mode of action. The antimicrobial drug-target interactions and their effects are well described [12,13,14]. In general, the four major mode of actions (MOAs) include interference with cell wall synthesis (e.g., penicillins), interference with nucleic acid synthesis (e.g., rifamycins), inhibition of metabolic pathways (e.g., sulfonamides), and inhibition of protein synthesis (e.g., erythromycins) [15,16]. A fifth MOA has been identified for the polymyxins and colistin, which disrupts the bacterial cell membranes by increasing membrane permeability [17].

Based on the success of antimicrobial drugs during the “Golden Age” of antibiotic discovery (1940s–1960s) some experts were confident that “the tide had turned” in the war against pathogens causing severe infections. However, microbes develop the ability to resist the effects of an antibiotic whenever the dose of the drug is too low to eliminate the whole population of the pathogen (minimum bactericidal concentration (MBC)) and thus, antibiotic resistance developed shortly after the drugs were applied [18,19,20,21,22,23,24]. Resistance to antibiotics arises from chromosomal mutations or acquisition of genetic elements encoding resistance genes (horizontal gene transfer). Several resistance mechanisms have been reported [25,26,27]. The most frequent resistance mechanisms are exported through the efflux pumps, degradation or inactivation of an antibiotic, and modification or alteration of the cellular target of the antibiotic [15,28]. Furthermore, the overuse and misuse of antimicrobial drugs have promoted and accelerated the spreading of antibiotic resistance (e.g., methicillin-resistant *Staphylococcus aureus* (MRSA) and vancomycin-resistant *enterococci* (VRE)). New resistance mechanisms are constantly being reported, and transmission elements are identified on a regular basis [29]. According to the World Health Organisation (WHO), antimicrobial resistance is widely regarded as one of the greatest global challenges to humanity (estimated 10 million lives a year by 2050) [30]. Thus, guidelines and initiatives are being put forward in order to limit the further spread of antimicrobial resistance [31]. Some of these initiatives include the development of vaccines, faster diagnostic tests to ensure appropriate antibiotic administration, and immune-based therapies [32]. These measures could provide promising solutions for prevention of infections however, once infection occurs in a human host, antibiotics remain the choice of treatment for bacterial and fungal infections. Therefore, the need for new antibiotic classes and improvement of the old compounds remain more important than ever. Unfortunately, discovery of novel antibiotic classes is halted by the low return on investment, forcing companies to abandon their discovery platforms. According to an issue brief of The Pew Charitable Trusts from March 2019 [33], out of the roughly 38 companies currently invested in antibiotic clinical development, only four of these are multinational pharmaceutical companies. Furthermore, 90% of the current products in development are studied by small pharmaceutical or biotech companies, which are ill-equipped to deal with the expense of possible setbacks, delays or even rejections, which are often faced in clinical trials [34]. 

Since polyketide-derived antimicrobials have had a historical significance in human therapy, this group of compounds remain important for continuation of research aiming at: the identification of new structures and activities, the biosynthesis and/or chemical semi-synthesis for discovery of novel derivatisation routes, and finally, at understanding of resistance mechanisms to overcome this obstacle. 

In this review, we focus on the therapeutically relevant polyketide drugs which were derived from actinomycetes. We describe compounds active against microbial pathogens, including pathogenic fungi and bacteria, their biosynthesis in the natural producer, and the most recent results on production optimization and derivatisation attempts. This review should serve as a “lexicon” of actinomycetes-derived antimicrobial polyketides, not only to scientists from the Natural Products field, but also to clinicians and others interested in this topic.

## 2. The Biosynthetic Assembly Lines

The biosynthesis of polyketides and nonribosomal peptides requires the PKS and NRPS enzymatic machineries. PKSs are grouped into type I, II, and III, and can be iterative (type I, II, III) or modular (type I) [35,36,37,38,39]. The diverse polyketide assembly lines have been previously reviewed [7,40,41,42,43]. Here, we introduce types of PKSs that are represented in this review. 

The modular type I PKSs can give rise to large, complex polyketides. A “minimal PKS module” is composed of three essential domains; the acyltransferase (AT), the ketosynthase (KS), and the acyl carrier protein (ACP). The AT domain selects and loads the building blocks onto the activated ACPs, while the KS domain catalyses the decarboxylative Claisen-like condensation of the newly loaded unit and the already existing polyketide chain. The β-keto-processing of the generated chain is accomplished by optional domains, such as the ketoreductase (KRs), the dehydratases (DHs) and/or the enoyl reductases (ERs). The final chain termination and release of the polyketide intermediate from the PKS is facilitated by the thioesterase (TE) domain, often found as the “last domain” in the multimodular PKS.

Type II iterative PKSs are responsible for the biosynthesis of aromatic polyketides [44,45,46]. The overall architecture of this type of PKSs appears simpler than the one of complex modular PKSs, as they only require the presence of three enzymes, the KS_α_, KS_β_ (also referred to as chain elongation factor (CLF)), and an ACP. During elongation, a thioester is bound to the ACP while the KS_α_KS_β_ complex orchestrates chain extension with malonyl-CoA units exclusively. The BGCs of type II PKSs often contain additional genes encoding cyclases (CYCs) and KRs, which act as chaperones and reductive enzymes to ensure the correct cyclisation of the polyketide precursor, respectively. Finally, the presence of aromatases (AROs) leads to the biosynthesis of aromatic ring systems. The iterative type I PKS [47,48] work in a similar fashion as the iterative type II PKSs [44]. However, the bacterial iterative type I PKSs only facilitate the biosynthesis of small aromatic polyketides [35,49]. 

NRPSs display a similar architecture to modular type I PKSs and function in an analogous manner [39,50]. The selection and loading of the building blocks (proteinogenic or non-proteinogenic amino acids) onto peptidyl carrier proteins (PCPs) of NRPSs is catalysed by an adenylation (A) domain, resulting in an amino acid–PCP–thioester. A condensation (C) domain attaches the amino acid to the so-far synthesised peptide (chain elongation). Modifications, such as epimerisation or N-methylation at the peptide chain may occur due to the activity of the optional epimerisation (E) and methyltransferase (MT) domain, respectively. The peptide chain is released from the NRPS by a TE domain. In the case of hybrid PKS/NRPS structures [51], the transfer of the growing precursor chain between the different megaenzymes might require additional enzymes. However, this remains speculative and requires deeper investigation.

## 3. Clinically-Relevant Polyketide Derived Antibiotics

### 3.1. Erythromycin and Derivatives

The history of the macrolide erythromycin began in 1949 with a soil sample, collected in Iloilo City, (Iloilo, Philippine Islands) by Dr. Abelardo Aguilar, who sent the sample to Lilly Research Laboratories, where the soil-dwelling bacterium *Saccharopolyspora erythraea* (formerly known as *Streptomyces erythreus*) and the antibiotic erythromycin were isolated [52]. Inspired by the name of the place from which the soil sample was collected, erythromycin was launched by Eli Lilly Co. in 1952 as Ilosone (also Ilotycin) (Table 1) for treatment of respiratory tract infections, skin infections, and Legionnaire’s disease. 

**Figure 2:**
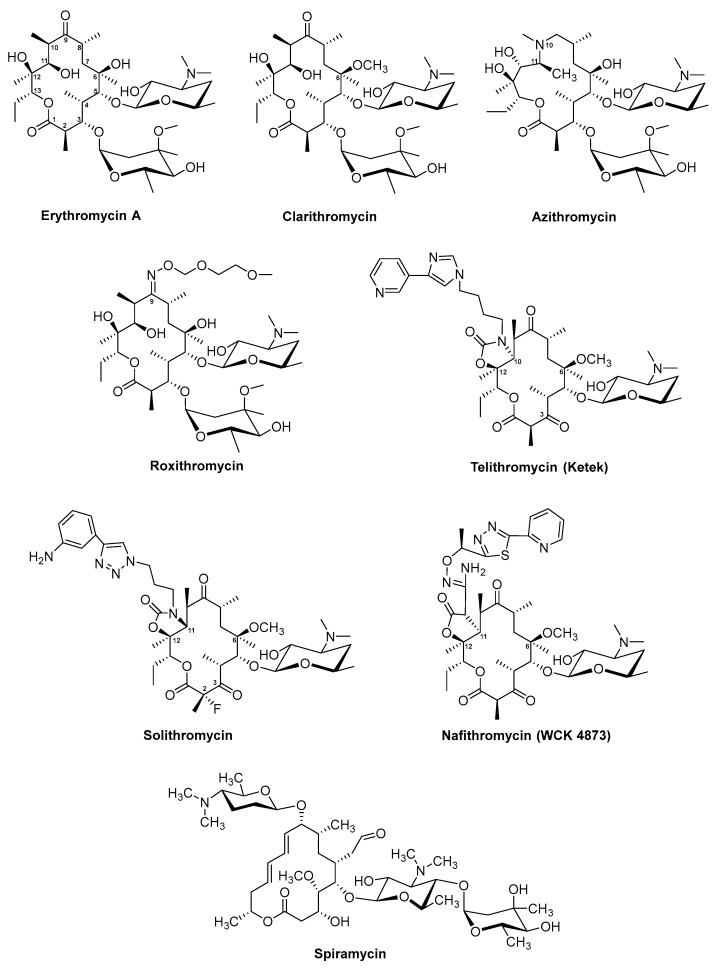
Structures of the two natural substances erythromycin A and spiramycin, and the semi-synthetic derivatives of erythromycin, including clarithromycin, azithromycin, roxithromycin, telithromycin (Ketek), solithromycin, and nafithromycin.

The antibiotic erythromycin is composed of a 14-membered lactone, erythronolide B, to which two deoxysugars l-mycarose and α-d-desosamine are attached [53,54] (Figure 2). The BGC of erythromycin remains one of the most-studied in terms of the type I PKS [55,56,57,58]. The precursor erythronolide B is generated by the modular type I deoxyerythronolide B synthase (DEBS), composed of the three enzymes DEBS 1–3, encoded by the genes *eryAI–eryAIII* (Figure S1). The PKS complex contains six modules, which are responsible for the initial loading of propionyl-CoA, the six steps of chain elongation with methylmalonyl-CoA as extender units, modification of the growing polyketide chain by reductive domains, and finally chain release and cyclization by the TE domain in DEBS 3 [57,59,60]. The hydroxylation of the released aglycone is catalysed by the cytochrome P450 enzyme EryF, resulting in the intermediate erythronolide B. The glycosyltransferases EryBV and EryCII/EryCIII attach the sugar structures, derived from thymidine diphosphate (TDP)-l-mycarose and TDP-d-desosamine, which leads to the production of erythromycin D. Erythromycin D is further converted to erythromycin B or C, which are the substrates for erythromycin A. Erythromycin B is formed whenever the methyltransferase EryG is acting on erythromycin D. In the case where the molecule erythromycin D is modified by the monooxygenase EryK, erythromycin C is produced as an intermediate. 

Although the strain produces a mixture of erythromycins, erythromycin A was the most abundant and biologically active compound [61]. Erythromycin shows activity against many Gram-positive and some Gram-negative bacteria, including *Haemophilus influenzae*, *S. aureus*, *Streptococcus pyogenes*, *Streptococcus pneumoniae*, *Legionella pneumophila*, *Neisseria gonorrhoeae,* and *Mycoplasma pneumonia* [61,62]. The antibiotic interacts with the large 50S subunit of the prokaryotic ribosomes and inhibits the protein biosynthesis [63,64]. More specifically, the antibiotic binds to the exit tunnel and blocks its large subunit whereby the elongation of the nascent peptide chain is stalled. This stalling leads to the premature dissociation of the peptidyl-transfer RNA (tRNA) from the ribosome [64,65]. As the inhibition occurs directly after initiation of protein synthesis, the nascent polypeptide chain remains short, however, the length size is determined by binding of the macrolide structure to the complex. Consequently, the size and conformation of erythromycin play a crucial role in the bioactivity of the molecule [66]. In case of erythromycin A, binding to the 50S subunit leads to dissociation and accumulation of peptidyl-tRNAs with six, seven, or eight amino acid residues. This accumulation causes depletion of the free tRNA pool, whereby protein biosynthesis is inhibited [65]. 

In order to improve the chemical, microbiological, and pharmacokinetic properties of macrolides, a lot of effort was put into the production of semi-synthetic and synthetic derivatives [63,170,171]. Some of the most successful semi-synthetic derivatives produced from erythromycin include the 14-membered clarithromycin and roxithromycin and the nitrogen-containing 15-membered azithromycin (Figure 2) [73,74]. Clarithromycin (trade name: Biaxin, Table 1) and roxithromycin (not commercially available, Table 1) were discovered in the 1980s in screening programs aiming at identification of erythromycin A variants with improved acidic stability and oral availability [74,75,172]. The pharmacokinetic profiles of erythromycin A and clarithromycin (6-*O*-methylerythromycin A derivative, Taisho Pharmaceutical Co.) are similar however, due to the methylation in clarithromycin, the antibiotic displays an increased and more stable absorption [62]. At a Croatian pharmaceutical company PLIVA, chemists succeeded in producing a range of novel erythromycin derivatives based on chemical modification of an erythromycin A oxime. One of the analogues was roxithromycin which contains a N-oxime side chain on the macrolactone ring [76,77,172]. However, since the general pharmacokinetic properties of roxithromycin are similar to those of erythromycin A, the search for more active and stable derivatives continued and ultimately, azithromycin (trade name: Zithromax, Table 1), which was the first azalide, was discovered [172,173]. Azithromycin (also CP 62,993 or XZ-450) differed from the other erythromycin derivatives due to its nitrogen-containing macrolactone ring [78,172,173]. In addition to its high stability and sustained tissue concentrations, azithromycin also had an extended activity spectrum, covering both Gram-positive and Gram-negative bacteria associated with respiratory tract infections [62,78,79,172]. Interestingly, azithromycin was the first derivative identified with a MOA different to that of erythromycin. Similar to other macrolides, azithromycin binds to the 50S ribosomal subunit. However, instead of one molecule, as it is the case for example for erythromycin, two molecules of azithromycin bind simultaneously to the 50S ribosomal subunit [174]. 

Another class of interesting semi-synthetic erythromycin A derivatives are the ketolides, which were developed as a part of a rational drug design approach [80,81,175]. Telithromycin (also HMR 3647) (Figure 2, Table 1), with the trade name Ketek, was the first ketolide introduced into clinical practice. The main difference between ketolides and other semi-synthetic erythromycin A derivatives is the exchange of the mycarose sugar moiety with a 3-keto group. Furthermore, telithromycin contains a large aromatic N-substituted carbamate side chain to which an imidazo-pyridyl group is attached at position C11/C12 of the aglycone structure [175]. Telithromycin was not only more stable than some of the other semi-synthetic erythromycin A derivatives under acidic conditions, the ketolide also retained activity against erythromycin resistance isolates of *S. aureus* and *S. pneumonia* [80]. It was suggested that the exchange of the sugar with a 3-keto group is the reason for the lack of macrolide–lincosamide–streptogramin B (MLS_B_) resistance against ketolides [176]. Furthermore, telithromycin binds to prokaryote ribosomes with ten-times higher affinity than erythromycin A [177]. Unfortunately, shortly after its introduction into the clinics, severe side effects were reported (“Ketek effects”). These included visual disturbance and hepatic failure [82]. As the reports of fatal liver failures in patients receiving Ketek increased, the US Food and Drug Administration (FDA) withdrew the approval of the drug for simple infections in 2007 [73]. 

Since many pharmaceutical companies were in possession of large macrolide and ketolide libraries, screening for novel macrolide drug candidates continued. Two novel ketolides, solithromycin (trade names: CEM-101 and T-4288) and nafithromycin (trade name: WCK 4873) were identified and used for treatment of community-acquired bacterial pneumonia (CABP) (Figure 2, Table 1) [73,83,84]. Solithromycin is metabolically stable and shows potency against CABP-associated bacteria and the Gram-negative pathogen *N. gonorrhoeae*, which is the causative agent of gonorrhea. However, the future of solithromycin is currently less certain. Despite promising phase III study results, the US FDA rejected the approval of the drug for treatment of CABP in 2016, stating their concerns for the lack of sufficient safety studies addressing the risk of hepatotoxicity [34]. 

At present, nafithromycin is the only ketolide in global clinical development [85]. The compound has been enrolled in a phase II clinical study for treatment of CABP. The structure of nafithromycin (Figure 2) differs from other ketolides. Instead of a carbamate at position C11/C12, nafithromycin contains a lactone group. In addition, the backbone of the side chain is differentiated by amino group and methyl group substituents. Finally, the side chain is attached at another position of the pyridine [73]. 

Erythromycin remains one of the best-studied 14-membered macrolides however, other naturally occurring macrolide antibiotics have been reported. These include oleandomycin (*Streptomyces antibioticus*, 1954), megalomicin (*Micromonospora megalomicea*, 1968), and pikromycin (*Streptomyces venezuelae*, 1951) [67,178,179], of which only oleandomycin made it into the clinics. Oleandomycin displays lower in vitro activity against *S. aureus* and *Streptococcus* spp. than erythromycin A [180]. However, scientists working at Charles Pfizer and Company claimed that the derivative sigmamycin (Table 1), a 2:1 mixture of tetracycline and oleandomycin, showed synergic effect on 22 *S. aureus* isolates, which were otherwise resistant to the two individual antibiotics [68]. Since other groups failed to reproduce the original findings, sigmamycin was removed from the market in the early 1970s [69].

Another naturally occurring macrolide is the 16-membered spiramycin (trade name: Rovamycin) (Figure 2, Table 1), which was discovered in 1954 from culture of *Streptomyces ambofaciens*, [70,71,181]. This was just two years after the discovery of erythromycin and due to a number of studies reporting better in vitro antimicrobial activity of erythromycin A compared to spiramycin, the latter never made it to clinical development in the US [72]. Although spiramycin is not commercially available in the US, pregnant women, suspecting an infection with the protozoan parasite *Toxoplasma gondii*, can request spiramycin for free from the US FDA after a consultation. The details are described in other comprehensive reviews [182,183].

Macrolide antibiotics such as erythromycin and its derivatives remain some of the most successful antibiotics in human therapy [73]. In addition to their application in treatment of common respiratory, genital, and skin infections, the overall properties of erythromycin and its derivatives still favour their use. These properties include, but are not limited to, good oral availability, narrow spectrum of activity, and a strong safety profile [73]. Furthermore, this compound class represents a safe and efficient alternative to patients suffering from allergies to penicillins [184]. 

### 3.2. Tylosin

**Figure 3:**
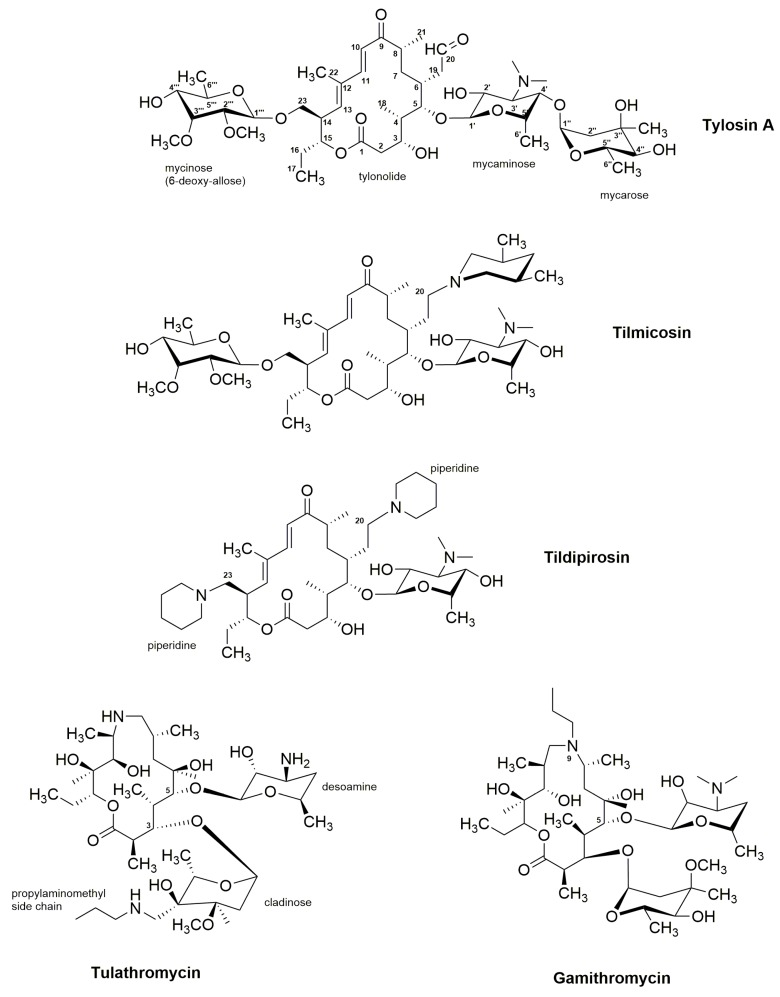
Structures of tylosin A and the four semi-synthetic derivatives tilmicosin, tildipirosin, tulathromycin, and gamithromycin.

The antibiotic tylosin (Figure 3, Table 1) was discovered in a preliminary screening by Denny and Bohrer in 1959 (Washington Research Laboratory, Washington, DC, USA) and later produced by Eli Lilly and Company in Indianapolis, Indiana (trade names: Tylocine, Tylan, tylosin tartrate) [86,185,186,187,188]. Tylosin was obtained from a fermentation culture of *Streptomyces fradiae* which was originally isolated from a soil sample collected in Thailand [86,189]. The compound is also produced by other streptomycetes strains such as *Streptomyces rimosus* [190] and *Streptomyces hygroscopicus* [191], however, *S. fradiae* remains the preferred choice for industrial fermentation of tylosin.

The naturally-derived tylosin is a mixture of the four 16-membered macrolide antibiotics tylosin A, B, C, and D [86,188,192,193,194,195]. Although tylosin A is found in highest concentration in the mixture (80–90%), tylosin B (desmycosin), tylosin C (macrocin), and tylosin D (relomycin) are also believed to contribute to the overall bioactivity of tylosin [195]. The structure of tylosin A (Figure 3) is a 16-atom polyketide lactone with the three deoxyhexose sugars d-mycaminose, mycinose (6-deoxy-d-allose), and l-mycarose attached [87].

The biosynthetic pathway of tylosin has been studied in detail [87,196,197,198,199,200]. With a size of ~85 kb, the tylosin (*tyl*) gene cluster occupies 1% of the entire genome of *S. fradiae*. Sequencing of the *tyl* gene cluster has revealed 13 genetic loci, *tylA* through *tylM*. These genes encode the PKS megaenzyme complex (TylGI–TylGV) for the assembly and cyclization of the precursor tylactone (Figure S2), proteins responsible for the biosynthesis of mycaminose, mycarose, and mycinose, hydroxylases, a methyltransferase, a reductase, and finally proteins involved in resistance and regulation. The biosynthesis of the tylosin polyketide chain is initiated by the loading of methylmalonyl-CoA to the first module in TylGI. The polyketide chain is extended by the attachment of four methylmalonyl-CoA, two malonyl-CoA, and one ethylmalonyl-CoA [88,201,202,203,204]. The TE-catalysed release of the aglycone and cyclisation of the structure yields the 16-atom tylactone, which is further modified at C20 and C23 by the two cytochrome P450 hydroxylases TylI and TylHI, respectively. The resulting intermediate tylonolide is then glycosylated through the attachment of the deoxyhexose sugars d-mycaminose, mycinose, and l-mycarose by TylMII, TylN, and TylCV, respectively [87,199,200,205] (Figure S2). Finally, to yield tylosin A, TylE and TylF modify the mycinose through the *O*-methylation of the C2‴ and C3‴ positions in the sugar. The remaining genes found in the *tyl* gene clusters encode ancillary proteins (crotonyl-CoA carboxylase/reductase (CCR), MetK, and, MetF), regulators (TylP, TylS, TylQ, TylU, TylR (TylT)), and proteins involved in resistance (TlrB–TlrD) [87]. 

Similar to other macrolides, tylosin inhibits protein biosynthesis in prokaryotes by binding to the large 50S subunit [88,89,185]. Poulsen et al. have shown that the disaccharide d-mycaminosyl-l-mycarose at the C5-atom in tylosin affects the binding of the molecule to the ribosome complex [206]. As opposed to erythromycin A (Figure 2), in which tri- and tetrapeptides are still released from the ribosome upon binding of the macrolide, for tylosin the C5-disaccharide extends binding of the macrolide to the ribosome, only allowing formation of dipeptides [206,207]. 

The antibiotic tylosin is approved exclusively for veterinary use. It is applied to treat leptospirosis, mycoplasmosis, and respiratory tract infection, caused by Gram-positive bacteria, including *Streptococcus* spp., *Staphylococcus* spp., *Clostridium perfringens*, and *Mycoplasma* spp. [208]. Historically, tylosin has been used as feed additive for cattle to prevent the development of liver abscesses and for swine as a growth promoter.

Tylosin remains a valuable antibiotic for use in veterinary medicine. However, derivatives of the native compound have been introduced in clinical practice as well. One such molecule is the semi-synthetic derivative tilmicosin, which has an extended antimicrobial spectrum compared to tylosin [90,91]. Tilmicosin displayed activity against *Pasteurella multocida* and *Pasteurella haemolytica*, and is important for treatment of bacterial pneumonia in young cattle. In addition, tilmicosin has shown improved activity against *P. multocida* in chickens. Tilmicosin is available under different trade names (Table 1), including Pulmotil and Micotil (Elanco Animal Health (https://www.elanco.com/), previously owned by Eli Lilly Company) and as Tilmovet (Huvepharma (https://www.huvepharma.com/)). Other semi-synthetic tylosin derivatives, which have been approved for treatment of respiratory diseases in animal production, include the 16-membered macrolide tildipirosin (trade name: Zuprevo) and the two 15-membered macrolides, tulathromycin (trade name: Draxxin), as well as gamithromycin (trade name: Zactran) (Figure 3, Table 1) [92,93,94,95]. 

In most cases, the 16-membered macrolides have less in vivo efficacy than the 14- and 15-membered macrolides. This is due to the fact that the side chains are metabolised and the generated products have a decreased activity compared to 14- and 15-membered macrolides.

### 3.3. Monensins

**Figure 4:**
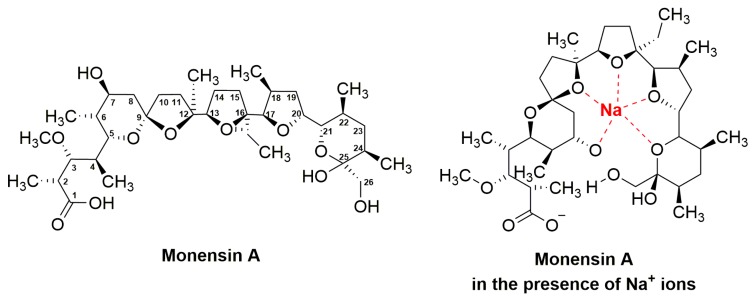
The structures of monensin A and monensin A in complex with sodium (Na^+^) ion.

The polyether ionophore monensin A (Figure 4, Table 1) (trade names: Coban, Rumensin, and Monensin) is another example of antibiotics being particularly important for veterinary medicine. The compound was identified in fermentation cultures of *Streptomyces cinnamonensis* and reported for the first time in 1967 as monensic acid by the Lilly Research Laboratories and the Department of Biochemistry of Indiana University School of Medicine [96,209]. In the natural producer, a mixture of monensin-like agents (monensin A, B and C) is synthesised, however, monensin A is the main constituent of this product (Figure 4). Monensin A is composed of five rings to which seven methyl, one ethyl, one carboxyl, and one hydroxyl group are attached [96]. Monensin B and C vary from monensin A by their side groups. In monensin B, the ethyl group on C16 is replaced by a methyl group, whereas in monensin C the methyl group at C2 is replaced by an ethyl group [210,211,212]. The carboxyl and hydroxyl groups can interact through hydrogen bonds, whereby a pseudocyclic conformation of monensin is formed (Figure 4). This results in an oxygen-containing center, which is selective for cations. Due to these conformational changes of monensin, an exterior composed of alkyl groups, which render the molecule hydrophobic and capable of passing the lipid bilayer of cell membranes, is built. The ionophore monensin A is able to bind monovalent metal cations such as Li^+^, Na^+^, K^+^, Rb^+^, Cs^+^, and Ag^+^. However, the compound prefers sodium cations for the formation of the ion–ionophore complex, which facilitates cation transport across cell membranes. This disturbs the natural Na^+^/K^+^ concentration gradient in Gram-positive bacteria causing death of the cell [97,210,213,214,215,216].

The genome sequencing of the producer and understanding the biosynthetic pathway of monensin have been crucial for optimizing the production of monensin in *S. cinnamonensis*. Sequence analysis of the 97 kb BGC of monensin (*mon*) has revealed a modular type I PKS, encoded by the genes *monAI–monAVIII* [98,217] (Figure S3). The first step in the assembly of the polyketide backbone is the loading of the “initiation module” of MonAI with malonate, which is derived from a malonyl-CoA starter unit. Twelve “downstream” extension modules in the PKS complex MonAI–AVIII catalyse the loading and condensation of additional four acetate, one butyrate, and seven propionate molecules, leading to the biosynthesis of the monensin polyketide chain. Since no TE domain is present in MonAVII, the release of the monensin intermediate must be facilitated by TEs acting in *trans*. Originally, it was assumed that either MonAIX and/or MonAX, which show homology to TEs involved in rifamycin, tylosin, and erythromycin biosynthesis [98], catalyse this reaction. Later, the enzyme MonCII, previously assigned as an epoxide hydrolase, was proposed as a putative novel TE, which might be responsible for the release of the monensin polyketide chain from the PKS assembly line [218]. The additional genes found in the *mon* gene cluster encode enzymes involved in isomerisation, epoxidation (MonCI), hydroxylation (MonBI, MonBII, and MonD), and methylation (MonE) of the structure to the final product monensin A [98,219]. Finally, the *mon* gene cluster includes three regulator genes, *monH*, *monRI*, and *monRII*, and the gene *monT*, which encodes an efflux protein, believed to confer self-resistance to the host [98,217,220,221]. 

Monensin A is used as a coccidiostat (inhibitor of coccidiosis) in poultry and as a non-hormonal growth promoter for cattle in the beef and dairy industry [97,210]. In addition, it was suggested that monensin A might also improve food metabolism in ruminants. Therefore, the feed additive Rumensin, which contains 6.6% monensin, was introduced for cattle to improve the composition of the intestinal bovine microbiota, reducing lactic acid formation, and ultimately leading to faster growth of cattle [99]. 

In 1971, monensin A was the first ionophore to be approved as a veterinary medicine. Two additional ionophore antibiotics lasalocid and salinomycin have subsequently been approved. Lasalocid, originally identified from fermentation broth of *Streptomyces lasaliensis*, was introduced in 1977 as a coccidiostat for poultry (trade name: Avatec) and later in 1982 as growth promoter for cattle (trade name: Bovatec). Salinomycin (trade name: Bio-Cox), produced by *Streptomyces albus*, received its approval as a coccidiostat for chickens in 1983 [210,222,223,224].

Given the nature of their applications, ionophore antibiotics have been subjected to increased concerns with regards to the association between their misuse as feed additives and development of resistance. However, studies on ionophore resistance in ruminal bacteria indicate that instead of resistance occurring as a result of mutations or horizontal gene transfer, it is more a matter of physiological selection [225]. Since ionophore antibiotics are restricted to only a few animal species and have never been used in human medicine, it appears that the risk of cross-resistance is rather low. Little is known about resistance development in bacteria subjected to ionophores. Based on in vitro and in vivo experiments several ionophore resistance mechanisms have been suggested. Those include the hypothesis of a proton-translocating enzyme which might counteract ionophore-dependent ion flux [226,227], the cell wall model of ionophore resistance [228,229,230], and the theory of extracellular polysaccharides (biofilm) [225], such as glycocalyx produced by ruminal bacteria. As no clear indications of resistance development and spreading have been reported, it is likely that the use of ionophore antibiotics as feed additives will continue [225]. 

### 3.4. Tiacumicin

**Figure 5:**
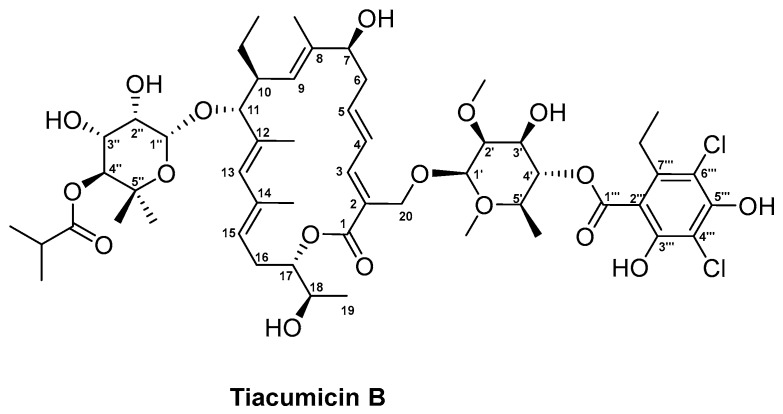
Structure of tiacumicin B.

In the 1980s, scientists working at Abbott Laboratories were screening soil samples for novel microbes with antimicrobial activities. In a soil sample from Hamden, Connecticut, USA, the novel Gram-positive bacterium *Dactylosporangium aurantiacum* subsp. *hamdenensis* subsp. Nov. AB718C-41 (NRRL 18085) was isolated based on its activity against MRSA. After scaling up the production, six novel compounds were isolated known collectively as the tiacumicins [100,231]. Structure elucidation of the tiacumicins further revealed a shared 18-membered macrolactone, which differed between the analogues based on the types of modification to the macrocyclic ring and the number and esterification pattern of the sugar groups attached. Out of the six compounds produced, tiacumicin B (Figure 5) was found in largest quantities [100].

The tiacumicin (*tia*) biosynthetic gene cluster of the original producer strains *D. aurantiacum* subsp. *hamdenensis* NRRL 18,085 was elucidated by Xiao and co-workers in 2011 based on sequence analysis and gene knockout studies [101]. The data revealed the presence of 50 open reading frames (ORFs) spanning an 110,663 bp DNA region. Additional sequence analysis has shown that 31 of the originally identified 50 ORFs are directly involved in tiacumicin biosynthesis. The remaining 83 kb DNA region includes genes encoding enzymes responsible for polyketide backbone assembly, sugar biosynthesis, glycosylation, halogenation, methylation, hydroxylation, epoxidation, and resistance determinants. The four genes *tiaA1* through *tiaA4* encode the multimodular type I PKS responsible for the assembly of the central aglycone tiacumicinone (Figure S4) [101,232]. After loading of the starter unit propionate, additional three malonates, four methylmalonates, and one ethylmalonate are condensed to the polyketide chain. Several genes are encoded in the *tia* BGC, which could provide the precursors for the polyketide. The synthesis of isobutyryl-CoA, propionyl-CoA, and acyl-CoA might be catalysed by TiaC and TiaD. Furthermore, the propionyl-CoA carboxylase encoded by *tiaL* might catalyse the formation of methylmalonyl-CoA from propionyl-CoA, and the CCR encoded by *tiaK* could be responsible for provision of the ethylmalonyl-CoA subunit [233]. Upon reaching the TE domain in module eight of TiaA4, the polyketide chain is released and cyclised, yielding the tiacumicinone precursor (Figure S4). An additional TE TiaE, which shows homology to the type II TE NysE involved in nystatin biosynthesis, is found in the *tia* BGC. Since these type II TEs are believed to act as repair enzymes, removing aberrant PKS precursors, TiaE has been hypothesized to have a similar role in *tia* biosynthesis [101,234]. Tiacumicinone undergoes several modifications including C18 and C20 hydroxylations, which are catalysed by the two cytochrome P450 enzymes TiaP1 and TiaP2, respectively, and the attachment of the rhamnose sugars at position C11 and C20, catalysed by the two glycosyltransferases TiaG1 and TiaG2 (Figure S4). The sugars are further modified. First, to yield the final 4-*O*-isobutyryl-5-methyl-β-rhamnose at position C11, the sugar C-methyltransferase TiaS2 and O-acyltransferase TiaS6 incorporate the methyl and isobutyryl moieties, respectively. The sugar at C20 also undergoes several modifications. The synthesis of the homo-orsellinic moiety is believed to be catalysed by an iterative type I PKS TiaB based on a propionyl-CoA starter unit and three malonyl-CoA extender units [101]. The transfer of the aromatic moiety to the C20 sugar could then be facilitated by the acyltransferase encoded by *tiaF* (Figure S4). Further modifications to the sugar are achieved by the actions of TiaS5, responsible for the 2′-*O*-methyltransferase reaction leading to 2-*O*-methyl-β-rhamnose and, finally, the dihalogenase TiaM, which is responsible for attachment of two chlorine atoms to the homo-orsellinic acid. Within the *tia* cluster, genes encoding proteins putatively involved in regulation and resistance are also found. This includes the putative LuxR class regulator TiaR1 and the putative ArsR family transcriptional regulator TiaR2, of which only the function of TiaR2 has been experimentally verified [101]. Since the Δ*tiaR2* mutant strain produced slightly higher levels of tiacumicin than the wild type strain, the regulator might act as negative regulator in *tia* biosynthesis. TiaT1 through TiaT4 show homology to membrane proteins and ATP-binding cassette (ABC) transporter and therefore, have been hypothesized to be involved in export of tiacumicin. However, genetic and biochemical studies are necessary to clarify their role in the biosynthesis of tiacumicin [101].

Coincidentally, in the same year as the tiacumicins were discovered and reported, a Japanese group, led by Satoshi Omura, reported the discovery of the antibiotics clostomicins, produced by *Micromonospora echinospora* subsp. *armenica* subsp. Nov [235]. Structure comparisons not only revealed tiacumicin B and clostomicin B_1_ to be identical, but also that a third antibiotic, lipiarmycin A3, discovered in 1975 from culture of *Actinoplanes deccanensis* A/10655, shared the same structure [231,236]. Ultimately, only tiacumicin B went through to further clinical studies. Although originally identified for its activity against MRSA, promising in vivo potency against *Clostridioides* (formerly *Clostridium*) *difficile* in animal models directed the development of tiacumicin B towards a narrow spectrum antibiotic for treatment of clostridia [233]. The antibiotic was approved by FDA in 2011 under the generic name fidaxomicin and trade name Dificid (Table 1) for treatment of *C. difficile* infections (CDI), which remains one of the main causes of hospital-acquired diarrhoea [233]. At the time, Dificid was regarded as an alternative to vancomycin, which was used to treat CDI. One of the major drawbacks of vancomycin is its broad-spectrum and bacteriostatic activity which promote reoccurrence of CDI. In contrast, fidaxomicin is a bactericidal, narrow-spectrum antibiotic and has been found to not only reduce recurrence of CDI but also promote survival of commensal gut microbes and prevent the development of colitis [102,237]. In addition, fidaxomicin is administered orally with no reports of resistance development in *C. difficile* isolates. In a recent US-based national survey, 1889 *C. difficile* isolates were collected from the stool of patients infected with the pathogen over the period of 2013 to 2016. The isolates were screened for susceptibility against fidaxomicin and showed that fidaxomicin MIC_50_ (maximum inhibitory concentration of an antibiotic, at which 50% of the isolates are inhibited) and MIC90 (maximum inhibitory concentration of an antibiotic, at which 90% of the isolates are inhibited) values toward *C. difficile* had not changed over the time period [238]. However, the fact that fidaxomicin was only recently introduced to the clinics stresses the need for continuous surveillance [238]. 

Recently, the MOA of tiacumicin B has been further clarified based on the cryogenic electron microscopy (cryo-EM) structure of *Mycobacterium tuberculosis* RNA polymerase (RNAP) holoenzyme in complex with lipiarmycin A3 [103]. Although the target of fidaxomicin was already known to be the bacterial RNAP [239], the recent findings could show the binding of the antibiotic to be specific for the RNAP clamp. Upon binding of fidaxomicin, the RNAP is trapped in an open-clamp conformation, which prevents the simultaneous engagement of the −10 and −35 elements needed for transcription initiation. Furthermore, since the binding sites of fidaxomicin do not overlap with those of other known RNAP inhibitors, including rifamycin and sorangicin, the risk of cross-resistance is lowered [103].

At the moment, fidaxomicin is the only substance approved for human therapy within the tiacumicin-like compounds. However, the advanced information on the structure activity relationship (SAR) of fidaxomicin might trigger a more rational structure-based design of drug analogues in the future [103]. 

The knowledge obtained from studies on the *tia* gene cluster aided the design of a dihalogenase-deficient Δ*tiaM* mutant strain, which produced 14 tiacumicin congeners of which 11 were novel derivatives [240]. Based on minimum inhibitory concentration (MIC) tests, tiacumicin congener 3 displayed higher potency against *Bacillus thuringiensis* (MIC = 0.5 μg/mL), *Micrococcus luteus* (MIC = 1 μg/mL), and *Enterococcus faecelis* (MIC = 4 μg/mL) compared to all other congeners and the parent compound tiacumicin B. Interestingly, in addition to its lack of chlorine atoms, congener 3 contained a propyl group on the aromatic ring. The four-fold antibacterial activity increase of congener 3 compared to tiacumicin B was putatively assigned to the structural change in the aromatic ring. In the future, this knowledge could provide the foundation for further gene manipulations, which would result in generation of novel tiacumicin derivatives with improved antibacterial activities [240]. Furthermore, these derivatives might also provide more cost-effective alternatives to fidaxomicin, the price of which remains a major point of criticism [241].

### 3.5. Rifamycin and Derivatives

**Figure 6:**
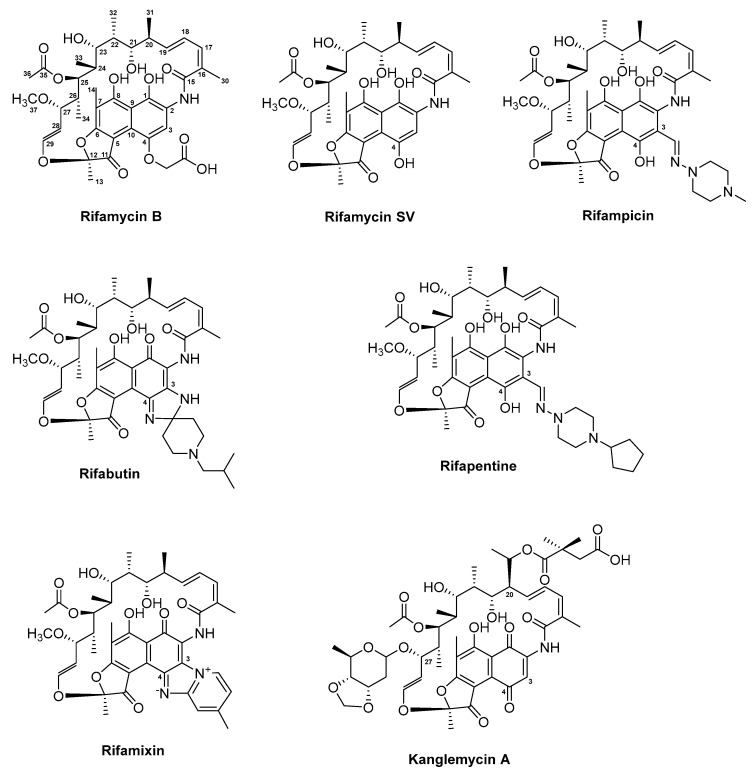
Structures of the two naturally derived substances rifamycin B and rifamycin SV and the semi-synthetic derivatives rifampicin, rifabutin, rifapentin, and rifamixin. Additionally, the structure of the natural substance kanglemycin A isolated from *Nocardia mediterranei* var. *kanglensis* 1741-64 is included.

Rifamycins (Figure 6) belong to the class of ansamycin antibiotics. This group of compounds is distinguished by a cyclic structure bridged at two nonadjacent positions by an aliphatic chain [104,105]. This basket or handle-like architecture gave rise to the naming ansamycins (Latin: ansa = handle). Ansamycins comprise a range of different substances. In the following, we cover rifamycin and its derivatives, which collectively belong to the group of naphtalenes-ansamycins. 

The history of this class of compounds started in 1959 with the isolation of the naturally-derived rifamycins, a complex mixture of several congeners produced by *Streptomyces mediterranei* [106]. More detailed studies on morphology, cell wall composition, and phage susceptibility resulted in re-classification of the strain, first as *Nocardia mediterranei*, and later as *Amycolatopsis mediterranei* [105,242]. Structure elucidation has revealed common architectures of the complex of rifamycins, which include a 17-membered aliphatic chain and a C–O bond linking the benzene ring to the chain [243] (Figure 6). In the initial attempts to separate and isolate each of the congeners from the fermentation broth of *A. mediterranei*, only rifamycin B, which constitutes 5–10% of the mixture, could be purified [244]. Since the substance was the least active congeners in the mixture, further studies were necessary to identify the more potent substances. One of the breakthroughs came with the elucidation of four additional rifamycin intermediates, including rifamycin S, L, O, and SV [105,245,246]. 

Using the sequence encoding RifK (3-amino-5-hydroxy benzoic acid synthase (AHBA synthase)) in the natural producer as a probe, the BGC of rifamycin was isolated from a cosmid library of *A. mediterranei* [107]. The gene cluster stretches over a 95 kb DNA region and contains 34 genes encoding all structural, modification, resistance, export, and regulatory elements. The assembly of the polyketide chain is governed by the modular type I PKS, encoded by the five genes *rifA* through *rifE* (Figure S5). Since the loading module within RifA contains domains with homology to A and T domains of NRPSs, the rifamycin assembly line is a hybrid PKS/NRPS [247]. The starter and extender units involved in the formation of the polyketide has been identified [248]. This includes the atypical starter unit AHBA, of which biosynthesis is orchestrated by enzymes encoded by *rifG* through *rifN*. The shikimate-related pathway of aromatic biosynthesis leading to the formation of AHBA was elucidated based on gene inactivation and heterologous expression [248]. Upon loading of AHBA, the polyketide chain is further elongated by the ten extender modules, which facilitate the addition of eight acetate and two propionate extender units [107,108]. The rifamycin PKS does not encode a typical TE domain. Instead, downstream of *rifE*, the gene *rifF* which shares homology to an arylamine *N*-acetyl-transferase is located. The role of RifF in chain release and macrolactam ring closure has been confirmed through an in-frame deletion, in which the Δ*rifF* mutant lost its ability to produce rifamycin B and instead accumulated shorter open-chain ketides [249]. An additional TE encoded by *rifR* has been identified in the rifamycin gene cluster [250]. However, since the Δ*rifR* and Δ*rifF*+Δ*rifR* mutants showed identical production profile, in which the same aberrant open-chain polyketides were detected, it was concluded that RifR is directly involved in the release of polyketide precursors from the assembly line. Furthermore, closer examinations of the PKS intermediates produced by the Δ*rifF* mutant revealed that while the tetraketide contained an unmodified aromatic chromophore, the pentaketide through the decaketide contained the bicyclic naphthoquinone moiety [251]. This indicated that naphthoquinone ring closure must occur while the polyketide chain is still attached to the PKS. This hypothesis was later confirmed based on the genetic studies of *rif19*, which encodes a 3-(3-hydroxyphenyl)propionate hydroxylase-like protein [252]. It was concluded that Rif19 catalyses the naphthoquinone ring closure, which occurs between module three in RifA and module four in RifB (Figure S5). Upon its release from the PKS, the polyketide precursor undergoes a series of post-modifications, including dehydrogenation at the C8 and hydroxylation of the C34, which results in the formation of one of the early intermediates in the post-modification pathway, namely rifamycin W [252,253]. The conversion of rifamycin W to rifamycin B involves the formation of the intermediate rifamycin SV (Figure S5) [105,252]. The final steps in the conversion of rifamycin SV to rifamycin B, through the intermediates rifamycin S, L, and O, have only recently been elucidated based on in vitro assays with the cytochrome P450 enzyme Rif16 and the two-subunit transketolase Rif15 (Figure S5) [254].

Additional genes encoding enzymes involved in regulation, export, and self-resistance have been identified in the rifamycin BGC. This includes the LuxR family regulator RifZ, which acts as an activator, and the TetR-family transcriptional regulator RifQ, which is a repressor of rifamycin biosynthesis in *A. mediterranei* [255,256,257]. The repressor RifQ is feedback-regulated by rifamycin B, the product of the rifamycin gene cluster in *A. mediterranei*. When a threshold concentration of rifamycin B is reached intracellularly, the substance interferes with the binding of RifQ to the promoter region of *rifP*, which encodes a membrane protein, whereby export of the antibiotic is initiated [258]. 

The importance of the rifamycins in human therapy is evident from their strong activities against a wide range of Gram-positive bacteria and to a lesser extent, some Gram-negatives. The reason for the higher MIC of Gram-negatives is most likely due to a reduced penetration of the rifamycins through the outer membranes of Gram-negatives [258]. The rifamycins remain the first-line treatment of tuberculosis (TB) caused by *M. tuberculosis* and for treatment of non-tuberculous mycobacterial infections [105,119,244]. Collectively, the rifamycins interact with the bacterial DNA-dependent RNAP whereby transcription is blocked. 

As previously mentioned, rifamycin B itself displays modest bioactivity and therefore, the need for derivatisation of the scaffold is necessary to produce rifamycin analogues with improved activity and availability. In fact, from the naturally derived rifamycins, only rifamycin SV has been introduced in human therapy. With a strong bioactivity against Gram-positives, including *M. tuberculosis*, and moderate activity against some Gram-negatives [244], rifamycin SV was introduced into the clinic in 1963 as a topical and parental agent. Extensive semi-synthesis efforts on rifamycins delivered several clinically relevant compounds. Using information gained from SAR studies, the first series of rifamycin analogous were generated based on substitutions at the C3/C4 positions in the aromatic moiety of the intermediate 3-formyl rifamycin SV. One of the resulting substances, 3-(4-methyl-piperazinyl-iminomethyl) rifamycin SV, coined rifampicin (Figure 6), displayed strong bioactivity against Gram-positive bacteria, including *M. tuberculosis* and *Neisseria* species, and modest activity against Gram-negatives [105,110]. Just as importantly, rifampicin was fat-soluble and could be administered orally. Ultimately, rifampicin (trade names: Rifadin and Rimactane) (Table 1) was introduced for human therapy in 1968 in Italy and in 1971 in the US, and has since then been an important agent in treatment of TB, leprosy, and other mycobacterial infections [243]. Additionally, rifampicin has also been an important substance for determining the MOA of rifamycins. Based on the 3.3 Å crystal structure of the RNAP core of *Thermus aquaticus* complexed with rifampicin, Campbell and co-workers determined the binding of the antibiotic to the β subunit within the DNA/RNA channel. This binding resulted in the physical blockage of the channel for the elongating RNA, when the transcripts reach a size of two to three nucleotides in length [111]. Determining the MOA of rifampicin also aided in understanding the high frequency of resistance development in rifamycin sensitive microorganism. Since pathogens can develop resistance to rifampicin with a rate of 10^−8^ to 10^−9^ per cell division, the application of the antibiotic is restricted to combination therapy, in which the antimycobacterial agent isoniazid is often used together with rifampicin, or used only in severe cases of TB infection [105]. The high rate of resistance is most commonly associated with the acquisition of mutations in the *rpoB* gene, which encodes the β subunit in the RNAP, whereby the binding affinity of the enzyme to the antibiotic is lowered. The *rpoB* gene is highly conserved in prokaryotes, however, pathogens acquire different levels of mutations in their respective *rpoB* genes, and consequently the level of resistance also can vary amongst isolates. For clinical isolates of *M. tuberculosis*, the three amino acids S411, H406, and D396 (*T. aquaticus* numbering) are most often mutated. Mutations at these positions in the RNAP core influence the hydrogen bonds formed between the hydroxyl groups on C8 and C21 of rifampicin, which interact with S411 and with H406 and D396, respectively [105,111]. 

Additional rifamycin analogues obtained through the derivatisation efforts on rifamycin SV have made it through to the clinics (Figure 6). One of these analogues is the 3-(((4-cyclopentyl-1-piperazinyl) imino) methyl) rifamycin S, also known as rifapentine (trade name: Priftin) (Table 1). Rifapentine was discovered during the initial derivatisation efforts on the C3/C4 aromatic moiety in rifamycin SV. The analogue was approved for treatment of TB in 1998 and similar to rifampicin can be administered orally [115,259]. Unfortunately, isolates which are resistant to rifampicin display cross-resistance to rifapentine, which has limited the application of the antibiotic.

The two rifamycin analogues rifabutin (trade name: Mycobutin) and rifaximin (trade names: Normix, Rifacol, Xifaxan) were approved for human therapy in 1992 and 2004, respectively (Table 1) [243]. Rifamixin ((4-deoxy-4)-methylpyrido [1),2)-1,2]imidazo-[5,4-c]rifamycin SV) is non-systemic and can reach high concentrations in the gastrointestinal (GI) tract, enabling its application for treatment of traveller’s diarrhoea caused by enteropathogens, including *Escherichia coli* [116]. More recently, rifaximin has been further implicated in the treatment and prevention of various GI diseases, including inflammatory bowel diseases such as Crohn’s disease and ulcerative colitis. Although the continuous administration of rifaximin has raised concerns for selection of resistance and undesirable disruption of the gut microbiota, several studies have shown that rifaximin may promote a healthy gut microbiota [117,260,261]. The beneficial effects of the antibiotic are lost quickly after the treatment with rifaximin was terminated which probably limits its application as a preventive agent for GI-associated diseases [118]. Only recently, an alternative antibiotic combination has been approved by the US FDA for the treatment of traveller’s diarrhoea. The oral formulation developed at Cosmo Pharmaceuticals N.V. (Ireland) is based on the unique combination of rifamycin SV together with the MultiMatrix (MMX^®^) technology, which ensures colonic delivery of the antibiotic [109]. Based on in vitro studies, rifamycin SV MMX^®^ (trade names: Aemcolo and Relafalk) (Table 1) was found to exhibit antibacterial activity against most enteropathogens associated with traveller’s diarrhoea. Moreover, in later phase III clinical studies, the oral substance was well-tolerated in patients undergoing treatment and could shorten the duration of non-dysenteric traveller’s diarrhoea [109].

The semi-synthetic derivative rifabutin (4-*N*-isobutylspiropiperidylrifamycin S) was approved for human therapy due to its inhibitory activity against a number of rifampicin-resistant *M. tuberculosis* clinical isolates in addition to its well-documented activity against *Mycobacterium avium* and *M. intracellulare* (also referred to as *Mycobacterium avium-intracellulare* complex (MAC)) [112,113]. MAC infection frequently occurs in patients with acquired immunodeficiency syndrome (AIDS) and when administered prophylactically as a 300 mg daily dose, rifabutin significantly reduces the incidence of MAC bacteraemia. However, due to the risk of cross-resistance between rifampicin and rifabutin in *M. tuberculosis* isolates, rifabutin is restricted to MAC patients and for newly diagnosed and multidrug-resistant TB in Europe [114].

During the derivatisation of rifamycins, modifications to the ansa bridge often led to compounds with lowered activities [262]. Recently, the natural ansamycin antibiotic kanglemycin A, which has an altered ansa chain, was found to maintain potency against rifampicin-resistant bacterial isolates, including multidrug-resistant *M. tuberculosis* (Figure 6). Kanglemycin A was originally isolated from *Nocardia mediterranei* var. *kanglensis* 1741-64 in 1988, however, the MOA of the compound was only recently elucidated. Based on the crystal structure of the RNAP-promoter complex of *Thermus thermophiles* with kanglemycin A, it became evident that the sugar (β-*O*-3,4-*O*,*O*′-methylene digitoxose) and acid (2,2-dimethyl succinic acid) moieties on the ansa bridge in the molecule increase the binding surface of the antibiotic with the RNAP. Furthermore, the additional interaction of the sugar group of kanglemycin A was hypothesized to limit the frequency of resistance development as this would require two simultaneous mutations in the binding pocket of the RNAP [119,120]. Although kanglemycin A (Table 1) itself has not been introduced to the clinics, its discovery might open up for future structural derivatisation efforts in which the ansa bridge might prove to be a valuable “target” for generating new synthetic ansamycin antibiotics with improved bioactivities.

### 3.6. Tetracyclines

**Figure 7:**
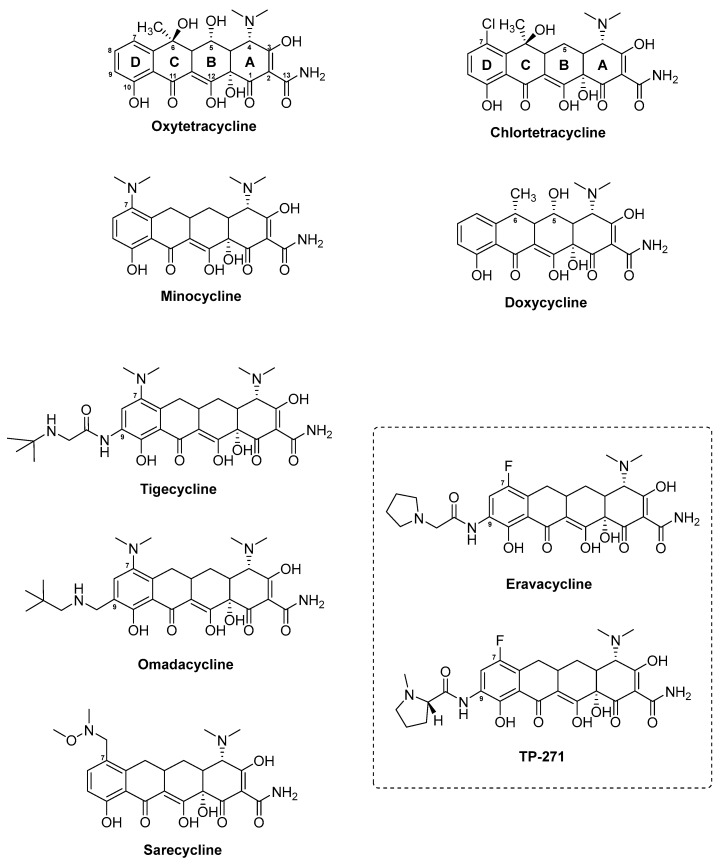
Structures of the naturally derived chlortetracycline and oxytetracycline and their semi-synthetic derivatives doxytetracycline, minocycline, tigecycline, omadacycline, and sarecycline. Eravacycline and TP-271 are fully synthetic tetracycline analogues.

The tetracycline group of antibiotics covers a variety of natural and semi-synthetic compounds. There are three naturally derived tetracyclines that have been described in detail and have formed the basis for 2nd and 3rd generation tetracycline derivatives. The three natural substances include chlortetracycline (trade names: Aureomycin, Table 1) discovered from the fermentation broth of *Streptomyces aureofaciens* in 1948 [125], oxytetracycline (trade name: Terracycline, Table 1) found in the broth of *Streptomyces rimosus* in 1950 [121], and finally tetracycline, which was discovered as a fermentation product of *Streptomyces viridofaciens* and the two aforementioned strains [263] (Figure 7). Based on the structural elucidation of the three compounds, which revealed a characteristic DCBA naphthacene tetra-cyclic core (Figure 7), the agents later became descriptively known as the tetracyclines [264]. Within the group of tetracyclines, tetracycline represents the simplest of the structures, whereas chlortetracycline and oxytetracycline contain modifications at the core cyclic structure. While chlortetracycline contains a chlorine atom at the C7 atom in ring D, oxytetracycline instead harbours a hydroxyl group at the C5 position in ring B.

The biosynthesis of chlortetracycline and oxytetracycline follows a similar logic and in the following, we focus on the biosynthetic principle of the latter compound. The BGC of oxytetracycline covers a 21.2 kb DNA region in *S. rimosus* and contains 21 ORFs encoding structural, modification, resistance, and regulatory elements [265]. The biosynthesis of oxytetracycline starts with the assembly of the polyketide precursor by the type II minimal PKS encoded by the genes *oxyA* through *oxyC* (Figure S6). More specifically, the KS_α_ (OxyA) and KS_β_ (OxyB) form the heterodimer responsible for chain elongation, while the ACP (OxyC) provides the extender units. An interesting feature of the natural tetracyclines is the unusual starter unit, malonamate, giving rise to the amide group on ring A in the naphthacene core [122,265]. The gene encoding the enzyme responsible for the provision of this starter unit has been identified as *oxyD*, which is located immediately downstream of the minimal PKS-encoding genes *oxyA* through *oxyC* in the oxytetracycline gene cluster in *S. rimosus*. The protein sequence of OxyD shows homology to the ATP-dependent class II asparagine synthases and it is believed to catalyse the transamination of malonate to yield malonamate [266]. Once attached to the minimal PKS, the malonamate is condensed with eight additional malonates, giving rise to the amidated decaketide backbone, which is released from the megaenzyme and further processed by AROs and CYCs encoded by genes in the oxytetracycline gene cluster (Figure S6). Based on the heterologous expression of the extended minimal PKS of oxytetracycline (OxyABCD) in *Streptomyces coelicolor* CH999, three enzymes have been identified and assumed to play a role in the correct cyclisation of the polyketide precursor [267]. This includes the NADPH-dependent KR OxyJ, which catalyses the C9 reduction in ring D, the two-component CYC/ARO OxyK which is responsible for closing the first ring (ring D), and the CYC OxyN, catalysing the ring closure of the second ring (ring C). An additional putative CYC, encoded by *oxyI*, has been identified in the oxytetracycline gene cluster, however, since the heterologous expression of OxyABCDJKNI displayed a profile similar to that of OxyABCDJKN, it was concluded that OxyI is not directly involved in cyclisation of the third or fourth ring (ring B and A, respectively). Instead, closing of these two rings is believed to occur spontaneously [268].

Upon ring closure, the stable intermediate pretetramid is formed, which undergoes a series of modification reactions, of which the actual sequence remains to be clarified [269] (Figure S6). The modification of pretetramid involves a C6 methylation in ring C, which is catalysed by the S-adenosyl methionine (SAM)-dependent methyltransferase OxyF, yielding the intermediate 6-methylpretetramide. In the next step, the dihydroxylation of C4 and C12a in ring A, yielding the substance 4-keto-anhydrotetracycline, is catalysed by the two oxygenases OxyL and OxyE. While OxyL can give rise to both hydroxylations, OxyE only catalyses the addition of the hydroxyl group at the C4 position. The reason for the extra function of OxyE seems to be a matter of increasing the rate of the enzymatic reaction [268]. The further conversion of 4-keto-anhydrotetracycline is achieved first by the actions of the aminotransferase OxyQ, yielding the intermediate 4-amino-anhydrotetracycline, which then serves as a substrate for the SAM-dependent methyltransferase OxyT, catalysing a *N*, *N*-dimethylation that gives rise to anhydrotetracycline. The final enzymatic reactions leading to formation of oxytetracycline from anhydrotetracycline was recently elucidated by Wang and co-workers (Figure S6) [270]. In this study, OxyS was confirmed to catalyse the two sequential hydroxylations of C6 and C5 in ring C and B, respectively. Furthermore, OxyR was shown to act as a F_420_-dependent reductase, which is responsible for the final C5a−C11a reduction affording oxytetracycline (Figure S6). In the case of chlortetracycline, an additional gene, *ctcP*, encoding a flavin-dependent halogenase, has been identified in the chlortetracycline BGC in *S. aureofaciens* [126]. CtcP yields the final product chlortetracycline based on the halogenation at position C7 in ring D (Figure S6). In addition to structural and tailoring genes, the BGCs of chlortetracycline and oxytetracycline include additional genes involved in resistance (*otrA* and *otrB*) and regulation (*otcG* and *otcR*). In the case of the oxytetracycline BGC, the two genes *otrA* and *otrB*, which encode proteins responsible for host resistance, are found at the flanking regions of the actual gene cluster. Based on sequence similarity, OtrA shows homology to TetM and is believed to provide resistance to *S. rimosus* by binding the ribosome and competing with the produced oxytetracycline. OtrB encodes a membrane protein and ensures the efflux of oxytetracycline from the cell [269,271]. The genes for the regulators OtcG and OtcR are also present in the flanking regions of the oxytetracycline BGC in *S. rimosus* [269,272,273]. OtcG, is a LuxR family transcriptional activator which was shown to provide positive regulation of oxytetracycline biosynthesis since an inactivation of *otcG* resulted in a mutant strain producing 40% less oxytetracycline than the wild type strain *S. rimosus* [272]. The overexpression of *otcG* did not yield a significant increase in production of oxytetracycline. OtcR was assigned to a *Streptomyces* antibiotic regulatory protein (SARP) activator of the oxytetracycline in *S. rimosus* [273]. Since a deletion of *otcR* completely abolished oxytetracycline production in the producer strain, the authors tested whether its overexpression would result in increased yields of the antibiotic. Indeed, the introduction two additional copies of *otcR*, under the control of a SF14 promoter, in *S. rimosus* increased the yields of oxytetracycline to 6.24 g/L (6.49 times higher than the yields of the parental strain) [273].

Tetracyclines are broad-spectrum antibiotics with activities against a range of microorganisms, including Gram-positive and Gram-negative bacteria, chlamydiae, mycoplasmas, and protozoan parasites [123]. They act as bacteriostatic drugs by binding to the 30S subunit of the prokaryote ribosome in a reversible fashion whereby protein synthesis is inhibited [132]. Furthermore, crystal structures of tetracycline in complex with both, the 30S subunit and 70S ribosome of *Thermus thermophiles*, have expanded the knowledge of the MOA of tetracyclines [124,274]. Based on the first X-ray structure of Brodersen et al., the primary binding site of tetracycline (Tet1) was found to be located between the head and shoulder of the 30S subunit, just above the transfer RNA (tRNA) binding site. When binding the A site, tetracycline act as a physical barrier for aminoacyl-tRNA so that the peptide chain cannot be elongated and protein synthesis is stalled [123,274]. Furthermore, the oxidised hydrophilic part of the tetracyclines appear to provide the main chemical interactions with the 16S ribosomal RNA (rRNA) and therefore, they are most important for the binding [275]. Another interesting property of the tetracyclines is their ability to chelate ions (Mg^2+^). In addition to the hydrogen bond interactions between the hydrophilic side of tetracycline and the nucleotides on the 16s rRNA, the Mg^2+^ ions, coordinated by ring A of tetracycline, seem to further strengthen the binding to the ribosome [274]. The ion chelating properties of tetracyclines also explain their broad spectrum, including activity against Gram-negatives. In order to enter the periplasm of Gram-negatives, tetracycline forms a complex with Mg^2+^ allowing the antibiotic to pass through the porins of the outer membrane. To diffuse through the cytoplasmic membrane, tetracycline dissociates from Mg^2+^ enabling its passage through to the cytosol. The chelating complex is restored inside the cytosol [132].

The emergence of tetracycline resistance in clinical isolates has led to many studies to clarify the driving forces behind it. To date, four mechanisms by which bacteria acquire resistance to tetracyclines have been identified. These include active efflux of the antibiotic, ribosomal protection proteins, antibiotic modification, and alteration of the antibiotic target site, i.e., the ribosome. More information on the basis behind these individual resistance mechanisms can be found elsewhere [123,127,132].

The discovery of the naturally derived tetracyclines immediately triggered studies on improving the overall performance of the antibiotics. This resulted in the so-called 2nd generation tetracyclines, two of which, doxycycline and minocycline (Figure 7), were marketed in 1967 and 1972, respectively [123]. The discovery of doxycycline was based on the structural observations that the C6 hydroxyl group of tetracyclines greatly impact the stability of the molecules while antibacterial properties are not affected [128]. Consequently, derivatisation at this site of the molecule was conducted. The first attempt to alter the functional group at the C6 position in oxytetracycline gave rise to 6-methylene-5-hydroxytetracycline (methacycline), which was further modified to 6-deoxy-5-hydroxytetracycline, later named doxycycline (trade name: Vibramycin, Table 1). On the other hand, minocycline was the result of studies on biogenesis mutants of *S. aureofaciens*, the producer of chlortetracycline [129]. Here, scientists working at the former Lederle Laboratories succeeded in isolating the precursor C6-demethyl-C7-chlorotetracycline (demeclocycline), which gave rise to the C6-demethyl-C6-deoxytetracycline (sancycline) precursor, that ultimately was converted to the final substance 7-dimethylamino-6-demethyl-6-deoxytetracycline (minocycline, trade name: Minocin, Table 1) [130,131]. Both doxycycline and minocycline are lipophilic molecules, which allow for oral absorption and thereby extend their applicability in the human therapy. Furthermore, they display improved antibacterial activities compared to tetracycline, including potent activities against Gram-negative bacteria such as *E. coli* and *Pseudomonas aeruginosa* and against the Gram-positive bacteria *S. aureus* and *E. faecalis* [127]. The importance of their introduction in human therapy was further strengthened by their activities against tetracycline-resistant isolates.

To ensure the continuous potential of the tetracyclines, a second round of derivatisation efforts, aiming at discovering novel tetracycline analogues with activities against both susceptible and tetracycline-resistant isolates, was initiated in the 1990s [123]. These efforts gave rise to the so-called glycylcyclines, also referred to as the 3rd generation tetracyclines, including tigecycline, omadacycline, and sarecycline (Figure 7) [127,133]. Tigecycline (trade name: Tygacil) was the first of the 3rd generation tetracyclines and was the result of the derivatisation of minocycline (Table 1) [134]. Compared to its precursor, tigecycline contains a *N*-alkyl-glycylamido group at position C9 in ring D. This structural modification resulted in a ~10–100-fold improved binding of tigecycline to the ribosome when compared to tetracycline [127,134]. Based on the crystal structure of *T. thermophilus* 70S ribosome bound to tigecycline, it has been shown that while the derivative binds the 30S subunit in the same manner as tetracycline, the interaction of tigecycline with the ribosome is greatly improved based on its additional *tert*-butylglycylamido group [274]. In cell-free translation assays tigecycline was capable of retaining its binding to the ribosome in the presence of the two resistance determinants TetM and TetO, confirming the activity of the derivative against tetracycline-resistant clinical isolates, which had acquired resistance based on ribosome protection proteins [135]. Tigecycline was approved by the US FDA in 2005 [132]. The current major limitation of tigecycline is its poor oral bioavailability and consequently the antibiotic is administered intravenously, giving rise to nausea and vomiting in the majority of patients [276].

Three additional 3rd generation tetracycline analogues have been approved by the US FDA for various applications in human therapy. Two of these, omadacycline (trade name: Nuzyra) and eravacycline (trade name: Xerava), bear a C9 substitution on ring D and have been licensed as broad-spectrum antibiotics, while the third, sarecycline (trade name: Seysara), was developed specifically as a narrow-spectrum tetracycline antibiotic for treatment of acne (Table 1). Omadacycline is an aminomethylcycline derivative of minocycline, which was isolated based on its superior activity against tetracycline-susceptible and -resistant bacteria, lack of toxicity, and the oral bioavailability [136,137,264]. Additionally, omadacycline shows improved activity against Gram-positive isolates compared to tigecycline and superior activity against Gram-negatives compared to eravacycline. This improved activity is believed to be a consequence of the C7 dimethylamino group and the C9 aminomethyl moiety in ring D of the molecule, since these modifications allow the antibiotic to evade the most common resistant determinants of efflux and ribosome protection [276]. The antibiotic was approved by the US FDA in October 2018 for the treatment of acute bacterial skin/skin structure infections, caused by a variety of bacteria, including methicillin-susceptible and -resistant strains of *S. aureus*, and for treatment of CABP, caused by *S. pneumonia* and methicillin-susceptible *S. aureus* [138]. Furthermore, omadacycline is currently undergoing safety and efficacy studies for its use in treatment of uncomplicated urinary tract infections. Eravacycline, developed at Tetraphase Pharmaceuticals, Inc., varies from the other clinical available 3rd generation tetracycline analogues with its fluorine atom at the C7 position and a pyrrolidinoacetamido group on the C9 of ring D (Figure 7) [139,140]. It displays activity against both Gram-positives and Gram-negatives and has been approved by the US FDA for treatment of infections caused by multidrug-resistant microorganisms, including carbapenem-resistant *Enterobacteriaceae*, MRSA, VREs, and extended spectrum β-lactamase (ESBL)-producing *Enterobacteriaceae* [141]. Following the same logic as for tigecycline and omadacycline, the altered functional groups on this fully synthetic tetracycline derivative was designed to evade tetracycline resistance determinants, which is evident from the retained activity of eravacycline against TetM-protected *E. coli* cells tested in an in vitro transcription/translation assay [140]. An additional fully synthetic tetracycline derivative, named TP-271 (Table 1), is currently being investigated by Tetraphase Pharmaceuticals, Inc. for its activity against pathogens causing CABP, anthrax, tularemia, and bubonic plague [143,144]. Similar to eravacycline, TP-271 is TetM-insensitive and can be used as an agent for bacterial isolates with this type of acquired resistance [143]. According to the pipeline of Tetraphase Pharmaceuticals, TP-271 is currently in phase I clinical testing [145]. The latest 3rd generation tetracycline to be approved by the US FDA, is the sarecycline, an aminomethylcycline with the unique and stable methoxy(methyl)aminomethyl modification at position C7 in ring D. Unlike the other tetracyclines, sarecycline has been approved as a narrow-spectrum antibacterial agent for the treatment of moderate to severe acne vulgaris caused by *Cutibacterium acnes* (formerly *Propionibacterium acnes*). Sarecycline was developed to meet the need for a safe treatment regime against acnes vulgaris, while simultaneously limiting resistance development of *C. acnes* [142].

### 3.7. Streptogramins

**Figure 8:**
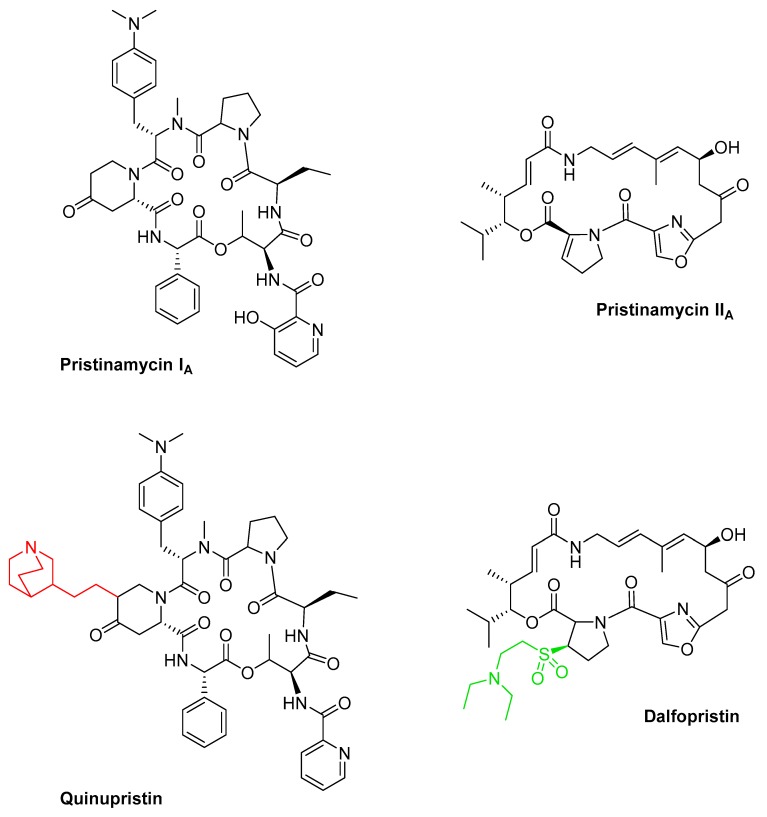
Structures of the two naturally derived antibiotics pristinamycin I_A_ and pristinamycin II_A_ and their two respective semi-synthetic analogues quinupristin and dalfopristin.

The group of streptogramins is an important class of antibiotics given their chemical complexity and potent antibacterial activity. They are composed of a mixture of two chemically unrelated substances, known as type A and type B streptrogramins, which are produced by the same host in a 70:30 ratio, respectively [277,278]. Streptogramin A and B, produced by *Streptomyces graminofaciens*, were the first in the group of streptogramin antibiotics to be discovered and provided the name for the substance class [279]. Following their discovery in 1953, several additional streptogramins were identified, some of which include mikamycin from *Streptomyces mitakaensis* [280,281], griseoviridin and viridogrisein from *Streptomyces griseoviridis* [282], as well as virginiamycin from *Streptomyces virginiae* [283]. Out of these substances, virginiamycin has had significant impact on veterinary medicine. Since its discovery, virginiamycin (trade names: Staphylomycin and Stafac) has been used all over the world in animal production as a disease control agent and feed additive for swine, poultry, and cattle. However, due to scientific concerns of resistance transmission between animals and humans, the use of drugs containing virginiamycin as a feed additive in Europe has been forbidden since 1999 [284]. Pristinamycin (Figure 8) is a close structural relative to virginiamycin. The antibiotic was first isolated from *Streptomyces pristinaespiralis*, which produces the two substances pristinamycin II_A_ and pristinamycin I_A_ in the ratio 70:30 [146,147]. Within the group of streptogramins, the two natural substances virginiamycin and pristinamycin and the semi-synthetic quinupristin–dalfopristin substance mixture (trade name: Synercid) (Figure 8, Table 1) have been subject to the most studies and will be the focus of this section. Since virginiamycin and pristinamycin share a common biosynthetic logic (Figure S7), only pristinamycin with its significant role in human medicine will be described.

The biosynthetic gene cluster of pristinamycin remains one of the largest bacterial antibiotic clusters identified, covering a DNA region of 210 kb [148]. Within this region, 45 genes, covering a 120 kb region, are involved in the biosynthesis, regulation, and resistance of pristinamycin. Pristinamycin II is synthesised by a hybrid PKS/NRPS complex responsible for the assembly of the starter unit isobutyryl-CoA with six malonyl-CoA extender units and the amino acids glycine, serine, and proline (Figure S7). The assembly starts at SnaE1, which ensures the successful loading of isobuturyl-CoA followed by its extension with two malonyl-CoA and glycine. The following four modules in SnaE2 and SnaE3 attach additional four malonyl-CoA extender units to the growing chain. In between the genes for the two megaenzymes SnaE2 and SnaE3, *snaG* through *snaL* are located. Together their respective enzymes are believed to be responsible for the C12 methyl group in pristinamycin II [148]. Downstream of *snaL*, the gene for the hybrid PKS/NRPS SnaE4 is located that is responsible for attachment of serine before transferring the precursor chain to the final module. The last NRPS module of SnaD, including the TE domain is responsible for the attachment of proline to the polyketide-hybrid chain and the release and cyclisation of the precursor chain. An interesting feature of the PKS is its lack of AT domains. Sequence analysis of the BGC and phylogenetic analysis of discrete ATs suggested that similar to the *trans*-AT KirCI, which is involved in kirromycin biosynthesis [285], SnaM also groups in the clade of discrete ATs of *trans*-AT-PKSs [148,286]. This indicates that SnaM might be responsible for an *in trans* loading of malonate units to assemble the pristinamycin II polyketide chain.

Pristinamycin I is synthesised from the NRPS encoded by genes *snbA*, *snbC*, and *snbDE*, which ensures the successful condensation of the four non-proteinogenic amino acids 3-hydroxypicolinic acid, l-aminobutyric acid, 4-*N*, *N*-dimethylamino-*N*-methyl-l-phenylalanine (DMAPA), 4-oxo-l-pipecolic acid, l-phenylglycine, and the two proteinogenic amino acids l-threonine and l-proline (Figure S7). Through the actions of the TE domain in the C-terminal of SnbDE, the precursor is released from the NRPS and cyclised. Additionally, 12 genes are involved in pristinamycin I biosynthesis through their synthesis of the amino acid precursors. This includes *hpaA* for 3-hydroxypicolinic acid, *pipA* and *snbF* for 4-oxo-l-pipecolic acid, and *papA/B/C/M* for DMAPA biosynthesis. For the non-proteinogenic amino acids, sequence analyses identified *pglA* through *pglE* to be involved in their biosynthesis [148]. Furthermore, within the 210 kb DNA region, several genes encoding regulatory elements have been identified [287,288]. Knowledge of the complex regulatory network behind pristinamycin I and II biosynthesis have fed metabolic engineering approaches in order to improve yields in the natural host [289,290]. In the study of Li and co-workers, the team systematically manipulated the cluster-situated genes *spbR* and *papR1* through *papR6* and found the best production enhancement of pristinamycin II_A_ when deleting either *papR3* or *papR5* and overexpressing both *papR4* and *papR6*. Surprisingly, strains overexpressing the major SARP activator PapR2 resulted in lowered pristinamycin production which could indicate a maximum threshold concentration of pristinamycin II_A_ in *S. pristinaespiralis* [290].

When applied separately as therapeutics, the type A and B streptogramins only provide a bacteriostatic effect. In combination, the two antibiotics act in a synergetic manner leading to a bactericidal activity against susceptible pathogens. Streptogramin antibiotics are active against a range of Gram-positive bacteria, including the severely resistant pathogens MRSA, vancomycin-resistant *S. aureus* (VRSA), *E. faecium* strains, and drug-resistant *S. pneumonia*. Activity against Gram-negative bacteria is mostly restricted to pathogens causing upper respiratory tract infection, including *H. influenza*, *Haemophilus parainfluenzae*, *M. pneumonia*, and *Moraxella catarrhalis* [150,277,278]. The synergistic activity of the type A and B streptogramins can be explained from the unique MOA, in that both antibiotics bind to the prokaryotic 50S ribosomal subunit however, at separate sites. The type A substances inhibit the early phase of protein elongation through their binding to the A and P sites on the 23S rRNA, thereby preventing the attachment of tRNA at each site [149,278]. Type B streptogramins also bind to the P site on the ribosome however, inhibit the late stage of polypeptide chain elongation by binding the exit tunnel of the ribosome. As a result, elongation of the nascent polypeptide chain is prevented and incomplete peptide chains are released from the complex. While the MOA of type B streptogramins is similar to that of erythromycin and related macrolides, the MOA for the type A streptogramins is similar to that of chloramphenicol. Furthermore, it has been shown that the binding of group A streptogramins to the P site leads to conformational changes in the subunit, which increases the affinity to the ribosome of the type B streptogramins. Even upon dissociation of the type A substances from the ribosome, the increase of ribosome affinity for type B streptogramins remains, explaining the synergetic effect of the mixture [291]. Recently, the crystal structure of the 50S ribosomal subunit from *Deinococcus radiodurans* in complex with Synercid (quinupristin/dalfopristin, Figure 8) has expanded our knowledge on the binding of streptogramins antibiotics to the ribosome [151]. Firstly, the two streptogramins share direct contact with a single nucleotide A2062 (*E. coli* numbering) through hydrophobic interactions and hydrogen bonds. This leads to conformational changes at A2062 whereby the binding of both antibiotics is strengthened. Secondly, an additional conformational change occurs when dalfopristin (type A) binds to the peptidyl transferase center (PTC) in that the universally conserved nucleotide U2585 is rotated to point away from the tunnel of the PTC. The altered conformation of U2585 in the PTC is stabilised by hydrogen bonds and might explain the post-antibiotic effect of streptogramin A antibiotics, in which protein synthesis is still inhibited after treatment with the antibiotic has been terminated [151].

To date, several resistance mechanisms against streptogramin antibiotics have been proposed. Some of these include target modification, drug inactivation, drug efflux, and impermeability [278,292]. Excellent reviews exist detailing the mode of drug resistance [292,293,294]. Briefly, a common resistance mechanism developed towards macrolide antibiotics is ribosomal target modification. This antibiotic resistance is encoded by the erythromycin-resistance methylase (*erm*) genes, which can mono- or dimethylate an adenosine residue of the 23S rRNA of the ribosomal 50S subunit [295]. Methylation changes the conformation of the ribosome and collectively causes resistance towards antibiotics of the MLS_B_ group. Additionally, the pathogen can develop resistance by drug inactivation through acetyltransferases (targets type A) and hydrolases (targets type B) or by drug efflux, encoded by genes such as *msrA*/*B*, *mefA*, *lsa*, and *vga* [294,296,297]. Resistance against streptogramins depends on the substance type. Type B streptogramins, which shares properties with erythromycin, are classified as MLS_B_ antibiotics, which suffer from cross-resistance between each other. Type A streptogramins instead show cross-resistance with lincosamides and pleuromutilins and group in the lincosamide–streptogramin A–pleuromutilin (LS_A_P) antibiotics. Since bacterial isolates with lowered susceptibility towards the synergetic mixture have already been isolated, it is evident that caution should be taken when administering these drugs. In order to reduce the risk of resistance towards this important class of compounds, streptogramins are classified as drugs of last resort [278].

Due to its hydrophobic nature, pristinamycin (Table 1) is administered orally, which limits its use in paediatrics and intensive care [152,278]. To solve the issue of bioavailability, medicinal chemists employed by the French chemical and pharmaceutical company Rhône-Poulenc Rorer worked on synthesising novel pristinamycin derivatives with improved water solubility. Their attempts proved successful and in 1999 the US FDA approved the antibiotic Synercid (Table 1), which is a 70:30 mixture of the two semi-synthetic derivatives dalfopristin (type A) and quinupristin (type B), for treatment of bacteraemia caused by VRE and skin/skin structure infections caused by methicillin-susceptible *S. aureus* and *S. pyogenes* [152,153,278]. Information on the synthesis of dalfopristin and quinupristin (Figure 8) remain scarce. From the published structures, it is known that quinupristin is the result of derivatisation of pristinamycin I_A_ at position five of the 4-oxo pipicolic acid residue and that dalfopristin is generated based on the substitution of pristinamycin II_A_ with a 2-diethylaminoethane thiol [292].

Synercid suffers from its own limitations, including a high treatment price and the risk of infusion site thrombosis in addition to myalgias and arthralgias [154]. A more recent example of a semi-synthetic pristinamycin derivative is NXL-103 (Table 1), which is a 70:30 mixture of flopristin (type A) and linopristin (type B) [154,155]. As opposed to Synercid, NXL-103 is administered orally thereby avoiding the complication associated with intravenous therapy. Additionally, NXL-103 has been shown to possess an improved activity against multiple Gram-positive isolates when compared to Synercid [155]. With its expanded activity against clinical Gram-positive isolates such as MRSA, methicillin-resistant *Staphylococcus epidermidis*, and VRE, NXL-103 could provide an additional treatment option to the current drugs on the market. However, since the report of phase II clinical trials in 2010, to the best of our knowledge, no new information on NXL-103 exists and the drug made no progress towards its introduction into the market [154].

## 4. Other Clinically Relevant Polyketide-Derived Antimicrobials

### 4.1. Nystatin A1

**Figure 9:**
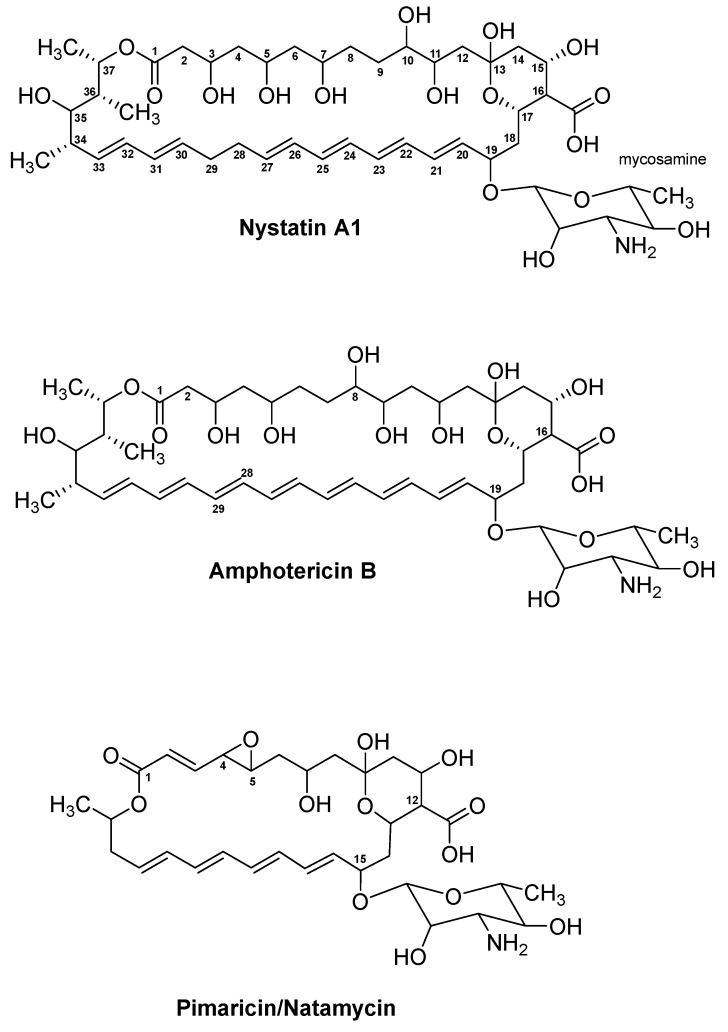
Structures of the three antifungal compounds nystatin A1, amphotericin B, and pimaricin (also referred to as natamycin).

The antifungal polyene macrolides are characterised by a large macrolactone ring containing 20 to 40 carbon atoms connected by a series of conjugated double bonds, an exocyclic carboxyl group, and a mycosamine sugar. Several compounds belonging to this group have been studied in detail, including candicidin, nystatin, amphotericin, and pimaricin (also referred to as natamycin) [298,299] (Figure 9). In this review, we focus on nystatin A1, amphotericin B, and pimaricin/natamycin, since these are the drugs still used in the clinic.

The first polyene macrolide was discovered in 1950 by E.L. Hazen and R.F. Brown from the New York State Department of Health and was the initially named fungicidin [156,300]. The compound was discovered from *Streptomyces* No. 48240 isolated from a soil sample collected at a farm owned by H. Nourse. Later, the strain was renamed *S. noursei* and the compound name changed from fungicidin to nystatin [157]. With the advances in analytical separation techniques it became evident that nystatin was a mixture of three components, namely nystatin A1, A2, and A3. Nystatin A1 was the major component in the fermentation mixture [301]. So far, *S. noursei* remains the commercial strain for production of nystatin, however, other strains, including *Streptomyces fungicidicus* ATCC 27,432 [302] and *Streptomyces albulus* ATCC 12,757 [303], have also been identified as nystatin producers.

Nystatin A1 (brand names: Mycostatin and Nystop) (Table 1) is composed of a 38-membered macrolactone ring which includes sets of two and four conjugated double bonds separated by one saturated bond (Figure 9). Similar to other polyene macrolides, nystatin contains a mycosamine sugar attached to the aglycone ring in addition to an exocyclic carboxyl group (Figure 9) [299,304].

The aglycone macrolide of nystatin (Figure 9) is assembled from one acetyl-CoA starter unit and further extended through condensation with three methylmalonyl-CoA and 15 malonyl-CoA extender units (Figure S8). The assembly of the nystatin precursor is governed by a type I PKS, composed of a total of one loading and 18 extender modules, which are all encoded in the six genes *nysA, nysB, nysC, nysI, nysJ*, and *nysK* [158]. The TE domain found at the C-terminal of module 18 in NysK is responsible for chain termination and cyclisation, forming the aglycone precursor of nystatin. Immediately downstream of *nysC*, the gene *nysE* is located. NysE displays protein similarity to TEs found in *Streptomyces venezuelae* and *Streptomyces fradiae*, however, the role of the enzyme has not been experimentally verified. It is postulated that this additional TE enzyme, like in the case of erythromycin and tylosin biosynthesis, acts as a “proof-reading” enzyme to avoid stalling at the PKS. Additional genes found in the nystatin gene cluster include the two P450 monooxygenases, encoded by *nysL* and *nysN*, which are responsible for the C10 hydroxylation and C16 methyl group oxidation, respectively. The role of NysN has been confirmed based on genetic inactivation. The Δ*nysN* mutant lost the ability to produce nystatin A1, and instead the analogue 16-decarboxy-16-methyl nystatin was isolated from the culture broth [160].

The biosynthesis and transfer of the mycosamine sugar to the aglycone of nystatin are carried out by three enzymes encoded by *nysDI*, *nysDII*, and *nysDIII* [158]. Specifically, the biosynthesis of the sugar is believed to be facilitated by the aminotransferase NysDII and the guanosine diphosphate (GDP)-mannose-4,6-dehydratase NysDIII, while the attachment of mycosamine at C19 of the nystatin aglycone is thought to be catalysed by the glycosyltransferase NysDI. Furthermore, it has been postulated that mycosamine is synthesised from a GDP-mannose instead of deoxythymidine diphosphate (dTDP)-glucose [298].

Two ATP-binding cassette (ABC) transporter-encoding genes *nysG* and *nysH* are located at the border of the nystatin gene cluster [305]. Since mutants in either of the two genes displayed similar phenotypes, NysG–NysH is thought to be part of the same transporter. Furthermore, four genes *nysRI* through *nysRIV* encoding regulator proteins have been identified and their role clarified in the nystatin gene cluster [299,306]. NysRIV is most likely directly controlling the expression of nystatin biosynthesis genes. More information on the regulation governing nystatin biosynthesis in *S. noursei* can be found elsewhere [299,306].

Nystatin A1 is primarily used as a topical agent e.g., in treatment of mucous membrane candidiasis caused by members of the yeast-like family *Candida* [307]. As it is the case for most polyenes, nystatin has a low water solubility and shows detectable toxicity, which restricts its application in human therapy. The explanation for the toxicity is found in the MOA shared among the polyenes. In general, the MOA of polyenes is based on their interaction with sterols in eukaryotic cell membranes, resulting in pores and increase membrane permeability for ions and small molecules, which is usually lethal for the cell [159]. Although nystatin displays a higher selectivity toward the ergosterol found in fungal cell membranes, it can also interact with the cholesterol of mammalian cells membranes, which is limiting its use for human therapy. Taken together, due to its undesired toxicity, low solubility, and lower antifungal potency than that of amphotericin B, the application of nystatin as human medicine remains restricted [160,308].

With the elucidation of the gene cluster responsible for the biosynthesis of the polyene macrolides candicidin, pimaricin, amphotericin, and nystatin it has become evident that the overall organisation of these cluster is highly similar [298]. This logic was utilised to design a polyene-specific polymerase chain reaction (PCR)-guided genome screening approach to screen for novel polyene-producing actinomycetes [309]. Using the sequence of a cytochrome P450 hydroxylase gene, which is similar between polyenes, the authors could identify and later confirm the presence of a Nystatin-like *Pseudonocardia* Polyene (NPP) gene cluster in the rare actinomycete *Pseudonocardia autotrophica* [310]. Structural analysis of NPP revealed an aglycone identical to that of nystatin however, with a modified sugar residue (a unique disaccharide moiety; mycosaminyl-(α1-4)-*N*-acetyl-glucosamine) [311]. Compared to nystatin A1, NPP A1 displayed a 300-fold increase in water solubility, 10-fold reduced hemolytic activity, but also ~50% lower antifungal activity against *Candida albicans*. The issue of lowered bioactivity was later solved through the manipulation of the ER domain in module five (ER5) of the NPP biosynthetic cluster [312]. Deleting this gene disables the reduction at the C28–C29 unsaturated bond in the aglycone of NPP A1 hence, generating a heptaene instead of the original tetraene. The new derivative NPP B1 displayed in vitro and in vivo activity against *C. albicans* and improved hemolytic activity compared to amphotericin B. However, the production yields of NPP B1 in the pathway-engineered strain (*P. autotrophica* ER5 mutant) were extremely low. In an attempt to solve this issue, the *P. autotrophica* ER5 mutant strain was subjected to *N*-methyl-*N*′-nitro-*N*-nitrosoguanidine (NTG) iterative random mutagenesis [312]. The resulting mutants were screened in zone-of-inhibition agar plug assays in which the mutant strain 3R-42 produced the largest inhibition zone. The transcriptional analysis further revealed a general up-regulation of the NPP biosynthetic genes in the 3R-42 mutant compared to the original ER5 mutant. Based on this observation, the authors introduced a second copy of each putative regulatory gene into the chromosome of the 3R-42 mutant strain and could determine a final NPP B1 production of 31.6 mg/L [312], which was a substantial increase in comparison to the 0.77 mg/L NPP B1 produced by the original ER5 mutant.

Additionally, studies on the biosynthesis of nystatin itself also open up for the discovery of novel derivatives with improved properties. In fact, based on genetic engineering of the nystatin gene cluster, Brautaset and colleagues obtained seven nystatin derivatives with altered exocyclic carboxy groups and polyol regions [160]. The mutational studies were based on the already obtained *S. noursei* mutant strain GG5073SP, in which the ER5 was deleted. This mutant produced a heptaene nystatin analogue, named S44HP. The introduction of a CL346AS mutation in the *nysN* of GG5073SP resulted in the mutant BSM1 and the isolation of a novel compound. Its structure was confirmed as 16-decarboxy-16-methyl-28,29-didehydro-nystatin (BSG005) by nuclear magnetic resonance (NMR) analysis. The authors also succeeded in generating a mutant strain, BSM3, which in addition to the mutation in *nysN* also was disrupted in the dehydratase (DH) domain in module 15, located in NysJ. The mutant strain BSM3 produced the analogue 5-oxo-5-deoxy-16-decarboxy-16-methyl-28,29-didehydro nystatin (BSG020). Both BSG005 and BSG020 display improved toxicities and comparable antifungal activities against disseminated candidiasis in a mouse model when compared to amphotericin B and thus, represent promising candidates for the development of new antifungal drugs [160]. The derivatives have not yet been introduced for human therapy however, based on information obtained from the homepage of the Swedish biotech company Biosergen AS, the company has selected the BSG005 candidate for further preclinical and clinical tests (Table 1) [161].

### 4.2. Amphotericin B

The antifungal polyene macrolide amphotericin B (trade names: Fungizone and Amphocin) was first discovered together with amphotericin A in the 1950s in the fermentation broth of soil-derived *Streptomyces nodosus* [162]. While amphotericin A contains a tetraene chromophore, amphotericin B possesses a heptaene (Figure 9).

The BGC of amphotericin B has been fully sequenced. The sequence analysis revealed that the cluster organisation is similar to the BGC of nystatin [313]. The polyketide chain is biosynthesised by an assembly line involving one loading (encoded by *amphA*) and 18 extension modules (encoded by the five genes *amphB*, *amphC*, and *amphI* through *amphK*) (Figure S8). Assembly of the precursor on the amphotericin PKS is initiated by the loading of a malonyl-CoA starter unit which is further elongated by additional 15 acetate and nine propionate extender units (Figure S8) [163]. In the last module, encoded by *amphK*, a TE domain is responsible for chain termination and release from the PKS. Two putative cytochrome P450 enzymes AmphL and AmphN are possibly involved in the modification of the amphotericin B structure. While AmphL most likely catalyses the C8 hydroxylation in the macrolactone, AmphN may facilitate the oxidation of the methyl group on C16 to yield a carboxyl group. Targeted deletion of *amphN* resulted in a *S. nodosus* mutant strain producing a amphotericin analogue in which the exocyclic methyl group is retained [314]. The fact that the antifungal activity of this analogue was unchanged, and the haemolytic activity reduced compared to amphotericin B, makes the derivative an interesting candidate for clinical studies.

Additional modification of amphotericin is facilitated by the glycosyltransferase, encoded by *amphDI*, which is responsible for the attachment of the mycosamine to the aglycone core of amphotericin [313,314]. The biosynthesis of the mycosamine is believed to be catalysed by a GDP-mannose-4,6-dehydratase encoded by *amphDIII*, which uses GDP-mannose derived from primary metabolism as substrate [298]. The product of the AmphDIII-catalysed dehydratase reaction is GDP-4-keto-6-deoxy-d-mannose and not GDP-3-keto-6-deoxy-d-mannose, which is the substrate recognised by the transaminase AmphDII. So far, no GDP-4-keto-6-deoxy-d-mannose-3,4-isomerase has been identified in any of the polyene gene clusters and it is hypothesised that the ketoisomerisation reaction is the result of a spontaneous, non-enzymatic reaction [298].

Export of amphotericin B has been hypothesised to be facilitated by two putative ABC transporters, encoded by *amphG* and *amphH*. The reason why two transporters are present in the gene cluster remains unknown. It has been speculated that ABC transporters can confer self-resistance of the producing host [313]. Based on the high degree of sequence similarity between nystatin and amphotericin PKS genes, it has been postulated that the genes *amphRI* through *amphRIV* are homologues of the genes *nysRI–nysRIV* in nystatin and encode regulatory proteins, which act in a very similar fashion [298,315].

Since its marketing in 1957, amphotericin B (Table 1) has been used as the “gold standard” for treatment of the most severe dimorphic fungal and yeast infections, caused by *Blastomyces*, *Candida*, *Cryptococcus*, and *Histoplasma* spp. [307]. The MOA of amphotericin B is identical to that of nystatin. Despite its preference for ergosterol found in fungal cellular membranes, amphotericin B also interacts to a lesser extend with the cholesterol found in mammalian cell membranes [164]. This and the side effects, including nephrotoxicity present a major limitation to the application of amphotericin B as an antibiotic for human therapy. In addition to its antifungal properties, amphotericin has also been implemented in delaying onset of prion disease in cultured cells with human immunodeficiency virus (HIV) and in inhibition of the protozoal parasite *Leishmania* [313].

Although amphotericin B shows a promising spectrum of activity and potential applications, the compound is poorly soluble in water and displays certain toxicity, which restricts its use in intravenous therapy. Consequently, only life-threatening fungal infections are treated with amphotericin B. Interestingly, despite its use as an antifungal drug for more than 40 years, reports of mycological resistance development in clinical fungi isolates against amphotericin B remain relatively scarce [316]. Nonetheless, resistance occurs, as it was demonstrated by the isolation of resistant *Candida* spp., *Fusarium* spp., and *Scedosporium apiospermum* [317]. In these fungi, drug resistance is most likely conferred by either the production of alternative ergosterols to which the amphotericin B is less efficient or simply by decreasing the ergosterol level in the fungal cell membranes. Both mechanisms reduce the potency of the antifungal drug [316].

The promising features of amphotericin B, such as broad-spectrum activity and low resistance against the compound were encouraging for numerous engineering attempts in order to improve the properties of the antifungal drug. A great improvement in the solubility of the amphotericin B was already achieved in the case of Fungizone, which is a mixture of amphotericin B and the bile acid deoxycholate [165]. Additionally, reduction in the overall toxicity of amphotericin B was further achieved through liposome encapsulation, which has resulted in the three formulations; Amphotec, AmBisome, and Abelcet [165]. Unfortunately, the reduced toxicity of the liposome-packed amphotericin B seems to come at a cost in antifungal efficiency.

The extensive investigations on amphotericin and polyene biosynthesis are of advantage for targeted engineering to increase production yields and to generate new derivatives with improved therapeutic properties. Furthermore, it should be noted that great efforts have been made in the field of semi-synthesis and several amphotericin B analogues with improved solubility and toxicity have been generated using different chemical approaches. Many of these have already been reviewed elsewhere [318]. Despite the many advances in both semi-synthesis and genetic engineering for obtaining amphotericin B analogues with improved properties, the yields of the derivatives are often very low, and to date, none of the reported amphotericin B analogues have made it through to the market. Consequently, the actinomycetes-derived substance amphotericin B remains one of the most important polyketides in the sparse portfolio of antifungal drugs. This urges the need for improved production titers in the natural producer strain. Recently, Zhang and co-workers set out to improve yields of amphotericin B in a newly isolated strain *Streptomyces* spp. ZJB 2013082, which produced the antifungal substance in low yields [319]. Using a combination of ultraviolet (UV) and NTG mutagenesis, the mutant strain N3 was isolated, which produced 1735 mg/L, a substantially increased amount of the product compared to the 56.2 mg/L obtained from the parent strain ZJB 2013082. Additionally, the N3 mutant accumulated less amphotericin A than the parent strain ZJB 2013082. This could be of industrial importance, since substance A is only allowed to account for more than five percent in the amphotericin mixture. The genome sequence of the N3 mutant is not yet published however, identification of the genomic architecture in the N3 mutant could help guide future engineering efforts to obtain a stable production host for amphotericin B.

### 4.3. Pimaricin/Natamycin

Pimaricin (later renamed natamycin, trade names: Natacyn and E235) (Figure 9, Table 1) was first discovered in the 1950s as the product of soil-derived *Streptomyces natalensis* isolated from the South African region of Natal [166,320]. Additional producer strains have been identified, including *Streptomyces chattanoogensis* [321,322] and *Streptomyces lydicus* [323].

The structure of pimaricin varies slightly from those of amphotericin B and nystatin A1 (Section 4.1) owing to the smaller size of the macrolactone (Figure 9). Pimaricin is composed of a 26-membered aglycone, containing four conjugated double bonds, to which the characteristic mycosamine sugar is attached at the C15 atom. The tetraene polyene further contains an exocyclic carboxyl group at C12, a functionally interestingly epoxide at C4/C5, and an internal hemiketal ring, which originates from a spontaneous cyclisation of the C9 keto group with a hydroxyl group on C13 [167].

The elucidation of pimaricin biosynthesis has relied primarily on genome sequencing and genetic studies of the two pimaricin-producer strains *S. chattanoogensis* and *S. natalensis* [320]. In the case of *S. natalensis*, an 85 kb-large genomic region containing 16 ORFs was identified from a cosmid library as the pimaricin gene cluster [167]. Pimaricin biosynthesis follows a logic, which is highly similar to that governing amphotericin B and nystatin biosynthesis. Since the gene clusters identified from *S. chattanoogensis* and *S. natalensis* are nearly identical, the following subsection will describe the studies on *S. natalensis*.

The assembly of the 26-membered lactone, termed pimaricinolide, is catalysed by a type I PKS composed of 13 (one starter and 12 extender) modules, encoded by the genes *pimS0–pimS4* (Figure S9). Chain initiation starts at PimS0 with the loading of a malonyl-CoA. Further elongation, catalysed by PimS1 through PimS4, leads to the condensation of additional 12 acetate units and one propionate unit to the growing polyketide precursor. In PimS4, the last domain, a TE, is responsible for the release and cyclisation of pimaricinolide [167]. Further examination of the pimaricin gene cluster has revealed an additional gene *pimI*, which encodes an enzyme with homology to the TE found in the candicidin gene cluster in *S. griseus* and to the TylO in the tylosin gene cluster in *S. fradiae*. It has been postulated that the additional TE in the pimaricin gene cluster helps to remove aberrant precursors from the PKS, ensuring continuous biosynthesis [167,204]. Upon its release from the PKS, pimaricinolide undergoes oxidation of the methyl group on C12 resulting in the formation of a carboxylic acid. This is catalysed by PimG, a cytochrome P450 enzyme. The resulting 12-carboxy-pimaricinolide is then glycosylated at the C15 hydroxyl group through the attachment of a mycosamine by the actions of the glycosyltransferase PimK. The final modification of the pimaricin precursor involves another cytochrome P450 (encoded by the gene *pimD*) which catalyse the oxidation leading to the spontaneous formation of an epoxy group between C4 and C5.

Sugar biosynthesis is believed to involve only two enzymes; PimJ, a GDP-mannose-4,6-dehydratase responsible for the conversion of GDP-mannose (from primary metabolism) to GDP-4-keto-6-deoxymannose, and PimC, a GDP-3-keto-6-deoxymannose aminotransferase, which synthesises GDP-mycosamine from GDP-3-keto-6-deoxymannose. Similar to what has been described for mycosamine biosynthesis in amphotericin B and nystatin, no gene encoding an enzyme responsible for the 3,4-isomerisation required for the conversion of GDP-4-keto-6-deoxymannose to GDP-3-keto-6-deoxymannose was found in the pimaricin gene cluster, and the reaction is believed to occur spontaneously [298,320].

The three gene products of *pimA*, *pimB*, and *pimH* have been putatively assigned to proteins ensuring the export of pimaricin in *S. natalensis*. While PimA and PimB group in the family of ABC transporters, PimH might encode an efflux pump [320,324]. The functions of PimA and PimB remain to be experimentally verified. For the homologues of PimA and PimB (ScnA and ScnB) in *S. chattanoogensis*, it was reported that they are involved in primary exporters of natamycin [324]. With amino acid sequence similarities of above 95% for ScnA/ScnB and PimA/PimB, it is likely that the later enzymes have a similar function and also act as primary transporters of pimaricin.

The regulatory mechanisms governing pimaricin biosynthesis has been studied in *S. natalensis* and *S. chattanoogensis*. Two transcriptional regulators PimM and PimR play an important role in pimaricin production in *S. natalensis* [325,326]. Furthermore, the amino acid exporter PimT and the putative cholesterol oxidase PimE add an extra layer of regulation to the pimaricin biosynthesis. While PimT was found to play a role in export of quorum-sensing pimaricin-inducer (PI) factor [327], PimE could act as a signalling molecule, triggering production of pimaricin in the producer in the presence of fungi [328,329]. The complex regulation cascade contains potential “targets” for engineering of the producer and increasing the production of the antifungal compound. Some of the most successful examples include overexpression of the regulator-encoding gene *scnRII* (homologue to *pimM*) in *S. chattanoogensis*, deletion of *sngR*, a γ-butyrolactone receptor-encoding gene, in *S. natalensis*, and chromosomal integration of the *Vitreoscilla* haemoglobin *vgb* gene in *S. gilvosporeus*, resulting in 460%, 460%, and 407% increase in pimaricin yields, respectively, in the mutant strains compared to wild types [320,330,331,332].

More than 40 years after the introduction of pimaricin to the market, it remains an important antifungal agent and it is still used in the treatment of fungal keratitis, an infection of the cornea caused primarily by filamentous fungi *Fusarium* and *Aspergillus*, and yeast-like *Candida* [168]. Due to its low water solubility and limited oral absorption, pimaricin is mainly available as a topical agent in human medicine. Recently, an antiprotozoan activity of pimaricin was detected which makes the compound attractive for potential treatment of keratitis caused by *Acanthamoeba*.

The MOA of pimaricin differs from that of nystatin and amphotericin B. While the main target of the pimaricin is ergosterol, which is the major sterol found in fungal cells membranes, pimaricin only binds to the lipid receptor. The interaction between pimaricin and ergosterol has been examined in *Aspergillus niger*, showing that upon its binding, pimaricin blocks transport of amino acids and glucose across the fungal plasma membrane, which leads to cell death [169]. Due to its specific interaction with ergosterol, the development of microbial resistance towards pimaricin is seen as posing only a minor risk [320]. This, combined with its low oral absorption, has paved the way for pimaricin as a food preservative. Sold under the label E235, pimaricin is approved as a protecting agent against yeast and mould and is used for surface treatment of hard, semi-hard, and semi-soft cheeses, and of dried, cured sausage in Europe. Other applications of pimaricin are summarised in the review by Aparicio and co-workers [320]. Additionally, pimaricin is the only antifungal agent to date, which has gained the generally regarded as safe (GRAS) status.

While pimaricin itself remains an important agent for treatment of fungal keratitis and as a food preservative, efforts to engineer strains which produce novel pimaricin analogues with improved solubility and toxicity have been undertaken. These efforts have been greatly aided by the complete genome sequencing of *S. natalensis* and *S. chattanoogensis*, both harbouring the BGC of pimaricin. Recently, Qi and co-workers could identify three novel pimaricin analogues based on a single mutation of the gene *scnG* in *S. chattanoogensis* (*pimG* in *S. natalensis*) [333], which encodes the cytochrome P450 enzyme responsible for the formation of carboxyl group at the C12 in pimaricin. Out of the three identified derivatives, 12-decarboxy-12-methyl pimaricin and 4,5-desepoxy-12-decarboxy-12-methyl pimaricin, both displayed reduced cytotoxicity compared to pimaricin. Additionally, 12-decarboxy-12-methyl pimaricin showed a two-fold increase in antifungal activity against *C. albicans* ATCC 14,053 compared to pimaricin. Through further biochemical and genetic analyses, 4,5-desepoxy-12-decarboxy-12-methyl pimaricin was found to be the precursor of 12-decarboxy-12-methyl pimaricin in the reaction catalysed by the C4/C5 epoxidase encoded by *scnD* (*pimD*). Therefore, to ensure the complete conversion of 4,5-desepoxy-12-decarboxy-12-methyl pimaricin into 12-decarboxy-12-methyl pimaricin, *scnD* was overexpressed in the Δ*scnG* mutant, which led to a 20% increase in production of the latter derivative. However, with an overall yield of 268 ± 10 mg/L for 12-decarboxy-12-methyl pimaricin in the best performing mutant, the needs for further engineering to optimise yields are necessary. In this case, the pathway-specific regulators PimM and PimR or the PI-factor could be the next targets for improving pimaricin derivative production [333].

## 5. Strategies and Tools for the Discovery of Natural Products

The emergence of antibiotic resistant microbes is alarming and underlines the urgent need for new drugs to combat the pathogens. However, the discovery and approval of new antibiotics is more difficult than expected [334,335,336]. Therefore, the question arises: how to improve the chances for finding new antimicrobial compounds? Recently, new approaches and advances of the existing technologies within the early stage of drug discovery and development were reported.

For natural product-derived antimicrobial compounds, the “journey” starts with the identification of the source (e.g., producer organism) and/or the bioactive molecule, responsible for the inhibition or killing of a pathogen. Already at this stage, the re-discovery rate of known structures might be reduced by taking samples and isolation of potential producers or compounds from undiscovered environments [337,338,339,340,341,342,343]. This is often limited by the fact that organisms originating from “extreme” habitats require special cultivation conditions and thus, many strategies were developed to overcome this barrier (e.g., co-cultivation [344,345,346], iChip [347,348,349,350,351,352], or combination of both [353]).

Confirmed or potential producers of new antimicrobial agents are further analysed by diverse “omics” approaches [342,354,355,356,357]. The downstream evaluation of the collected data sets using bioinformatics tools enables for example the identification of the BGC for the product of interest and/or provides an overview on the overall biosynthetic potential of target strain (genome mining) [9,358,359,360]. In cases where promising BGCs were identified, however, no products were found with the available fermentation and analytic methods [361,362,363,364], the expression of the clusters and production of the respective metabolite might be achieved by addition of elicitors [341,342,365,366,367,368,369] or the heterologous expression of the BGC in optimized hosts [370,371,372,373,374,375].

In order to improve the production of relevant products, including polyketides, molecular biology tools and methods (e.g., vectors, plasmids, recombinases, CRISPR-Cas9, promoters, and other synthetic parts as well as methods for their delivery (conjugation, protoplast transformation, and direct transformation)) are used. They play an important role for engineering of both, the natural producer and heterologous hosts. Challenges and new opportunities for the genetic manipulation of actinomycetes were recently reviewed [376,377,378,379,380,381].

Although each one of these cutting-edge technologies and approaches already contributed to the identification of new compounds, the interplay of the different disciplines will grant a better access to novel natural products with valuable bioactivities.

## 6. Conclusions and Outlook

Actinomycetes are one of the most prolific sources of biologically active secondary metabolites, including polyketides. In the past decades, numerous polyketide compounds were isolated and developed to highly potent antimicrobial drugs, which have saved millions of lives. However, rapid emergence of multidrug resistant pathogens is occurring worldwide which poses a severe threat to human health. This calls for the discovery and development of new antibiotics and antimicrobial strategies. Unlike drugs used in case of chronic diseases (e.g., diabetes, cardiovascular disease, cancer, arthritis, asthma), antibiotics are taken for a short period of time and thus, they are non-profitable and economically unattractive. This and several other obstacles such as high costs of the research and development and insufficient investment from stakeholders has prompted the big pharmaceutical companies to terminate the development of new antibiotics. Currently, it seems that the screening and development of new lead structures for novel antimicrobial agents is mainly conduced at public research and non-profit institutions.

The fact that many habitats around the globe are unexplored and poorly investigated for the presence of antibiotic-producing microbes, such as actinomycetes, motived researchers to collect samples from these environments and isolate the diverse producers of potentially new bioactive compounds. The valuable knowledge obtained from the investigation of the biosynthesis, regulation and natural resistance in the natural host of the old drugs as well as the recent developments within screening and isolation methods, sequencing and genome mining, and analytics potentiate the platforms for drug discovery. For example, the analysis of a relatively underexplored genus of *Actinoallomurus* led to the discovery of two new spirotetronate polyketide antibiotics NAI414-A and NAI414-B [382].

As exemplified in this review (Section 3.1), the success in derivatisation and combination of existing compound classes has enabled the continuous efficient treatment of otherwise resistant clinical isolates. In particular, the knowledge gained from detailed MOA and SAR studies of the antimicrobials have paved the way for the development of drugs with improved pharmacokinetic properties and expanded spectrum of bioactivity, compared to the original substance. In the future, semi-synthesis will continue to play an important role in drug development, of both, old and new drug candidates.

Last, but not least, support from governments and cooperation across the world e.g., public research institutions as well as United Nations organisations, the WHO, the Food and Agriculture Organization, and the inter-governmental World Organisation for Animal Health, combined with strategies offering long-term incentive for the pharmaceutical companies to reinvigorate their antimicrobial drug discovery platforms are an important political aspect that has been gaining more attention these days. The option of the US FDA to gain a fast-track approval of drug leads which can be used to treat serious or life-threatening conditions might further enhance the chances of taking on the expenses associated with drug discovery by pharmaceutical companies. The combination of all these efforts may give a competitive advantage in the never-ending race between the discovery of antimicrobials and the rise of drug resistance in pathogens.

## Figures and Tables

**Table 1:** Examples of actinomycetes-derived antimicrobial polyketide drugs.

Compound Class	Natural Product (NP) or Synthetic (S)	Compound Name	Trade Names	Original Producer Strain/Origin	Target/MOA	Most Relevant References
**Erythromycin and related compounds**	NP	Erythromycin A	Ilosone	*Saccharopolyspora erythraea*	Prokaryotic 50S ribosomal subunit	[52,55,58,61]
		Oleandomycin	Sigmamycin (with tetracycline)	*Streptomyces antibioticus*		[67,68,69]
		Spiramycin	Rovamycine	*Streptomyces ambofaciens*		[70,71,72]
	S	Clarithromycin	Biaxin	Erythromycin A derivative		[62,73,74,75]
		Roxithromycin	–			[76,77]
		Azithromycin	Zithromax	Erythromycin A (azalide)		[62,78,79]
		Telithromycin	Ketek	Erythromycin A (ketolide)		[80,81,82]
		Solithromycin	Solithera, CEM–101, T–4288			[83,84]
		Nafithromycin	WCK 4873			[83,84,85]
**Tylosin and derivatives**	NP	Tylosin A	Tylocine, Tylan	*Streptomyces fradiae*	Prokaryotic 50S ribosomal subunit	[86,87,88,89]
	S	Tilmicosin	Pulmotil, Micotil, Tilmovet	Tylosin A		[90,91]
		Tildipirosin	Zuprevo			[92,93]
		Tulathromycin	Draxxin			[94]
		Gamithromycin	Zactran			[95,96]
**Monensin A**	NP	Monensin A	Coban, Rumensin, Monensin	*Streptomyces cinnamonensis*	Ionophore (transport of Na^+^ ions)	[97,98,99,100]
**Tiacumicin B**	NP	Tiacumicin B	Dificid	*Dactylosporangium aurantiacum* subsp. *hamdenensis*	RNA polymerase σ factor	[101,102,103,104]
**Rifamycin and derivatives**	NP	Rifamycin SV	Aemcolo, Relafalk	*Amycolatopsis mediterranei*	Bacterial DNA–dependent RNA synthesis	[105,106,107,108,109]
	S	Rifampicin	Rifadin, Rimactane	Rifamycin SV		[106,110,111]
		Rifabutin	Mycobutin			[112,113,114]
		Rifapentine	Priftin			[115]
		Rifamixin	Normix, Rifacol, Xifacan			[116,117,118]
	NP	Kanglemycin A	–	*Nocardia mediterranei* var. *kanglensis*		[119,120]
**Tetracyclines**	NP	Oxytetracycline	Terracycline	*Streptomyces rimosus*	Prokaryotic 30S ribosomal subunit	[121,122,123,124]
		Chlortetracycline	Aureomycin	*Streptomyces aureofaciens*		[123,124,125,126]
	S	Doxycycline	Vibramycin	Chlortetracycline		[123,127,128]
		Minocycline	Minocin	Oxytetracycline		[129,130,131]
		Tigecycline	Tygacil	Minocycline		[132,133,134,135]
		Omadacycline	Nuzyra	Minocycline		[133,136,137,138]
		Eravacycline	Xerava	Fully synthetic		[139,140,141]
		Sarecycline	Seysara	Tetracycline		[127,133,142]
		TP–271	TP–271	Fully synthetic		[143,144,145]
**Pristinamycins and derivatives**	NP	Pristinamycin I_A_/II_A_ (PI_A_ and PII_A_)	Pyostacine	*Streptomyces pristinaespiralis*	Prokaryotic 50S ribosomal subunit	[146,147,148,149]
	S	Quinupristin (30%)/Dalfopristin (70%)	Synercid	PI_A_ and PII_A_ derivatives		[150,151,152,153]
		Linopristin (30%)/Flopristin (70%)	NXL–103	PI_A_ and PII_A_ derivatives		[154,155]
**Nystatin and derivative**	NP	Nystatin A1	Mycostatin, Nystop	*Streptomyces noursei*	Lipid receptor (ergosterol)	[156,157,158,159]
	NP	BSG005	–	*Streptomyces noursei* GG5073SP		[160,161]
**Amphotericin**	NP	Amphotericin B	Fungizone, Amphocin	*Streptomyces nodosus*		[162,163,164,165]
**Pimaricin/Natamycin**	NP	Natamycin	Natacyn, E235	*Streptomyces natalensis*		[166,167,168,169]

## Supplementary Materials

The following are available online at https://www.mdpi.com/2079-6382/8/4/157/s1, Figure S1: The erythromycin biosynthetic pathway, Figure S2: The tylosin biosynthetic pathway, Figure S3: The monensin biosynthetic pathway, Figure S4: The biosynthetic pathway of tiacumicin, Figure S5: The biosynthetic pathway of rifamycin, Figure S6: The biosynthetic pathway of oxytetracycline and chlortetracycline, Figure S7: The biosynthetic pathways of pristinamycin II and pristinamycin I, Figure S8: The biosynthetic pathways of nystatin and amphotericin B, Figure S9: The biosynthetic pathway of pimaricin.

## Abbreviations

A	adenylation domain	MBC	minimum bactericidal concentration
ABC	ATP-binding cassette	MIC	minimum inhibitory concentration
ACP	acyl carrier protein	MLS_B_	macrolide–lincosamide–streptogramin B
AHBA	3-amino 5-hydroxybenzoic acid	MOA	mode of action
ARO	aromatase	MRSA	methicillin-resistant *Staphylococcus aureus*
AT	acyltransferase	MT	methyltransferase domain
BGC	biosynthetic gene cluster	NADPH	nicotinamide adenine dinucleotide phosphate
C	condensation domain	NGST	Next Generation Sequencing Technologies
CABP	community-acquired bacterial pneumonia	NMR	nuclear magnetic resonance
CCR	crotonyl-CoA carboxylase/reductase	NRPS	nonribosomal peptide synthetase
CDI	*Clostridioides difficile* infection	NTG	*N*-methyl-*N*′-nitro-*N*-nitrosoguanidine
CLF	chain elongation factor	ORF	open reading frame
CoA	coenzyme A	PCP	peptidyl carrier protein
cryo EM	cryogenic electron microscopy	PCR	polymerase chain reaction
CYC	cyclase	PKS	polyketide synthase
DEBS	deoxyerythronolide B synthase	PTC	peptidyl transferase center
DH	dehydratase	RNA	ribonucleic acid
DNA	deoxyribonucleic acid	RNAP	RNA polymerase
dTDP	deoxythymidine diphosphate	rRNA	ribosomal RNA
E	epimerisation domain	SAM	S-adenosyl methionine
ER	enoyl reductase	SAR	structure activity relationship
ESBL	extended spectrum β-lactamase	SARP	*Streptomyces* antibiotic regulatory protein
FDA	Food and Drug Administration	Spp.	Species
GDP	guanosine diphosphate	TB	Tuberculosis
GI	gastrointestinal	TDP	thymidine diphosphate
Kb	kilobase	TE	Thioesterase
KR	ketoreductase	tRNA	transfer RNA
KS	ketosynthase	VRE	vancomycin-resistant *Enterococci*
LS_A_P	Lincosamide–streptogramin A–pleuromutilin	VRSA	vancomycin-resistant *Staphylococcus aureus*
MAC	*Mycobacterium avium-intracellulare* complex	WHO	World Health Organisation
