# Supplementary material for: Actinomycete-Derived Polyketides as a Source of Antibiotics and Lead Structures for the Development of New Antimicrobial Drugs
Source: Antibiotics (Basel). 2019 Sep 20;8(4):157. doi: 10.3390/antibiotics8040157 (PMC6963833; doi:10.3390/antibiotics8040157)
Supplement: Supplementary file 1 [file antibiotics-08-00157-s001.pdf]

Supplementary Material

Biosynthetic pathways for relevant actinomycete-derived polyketides

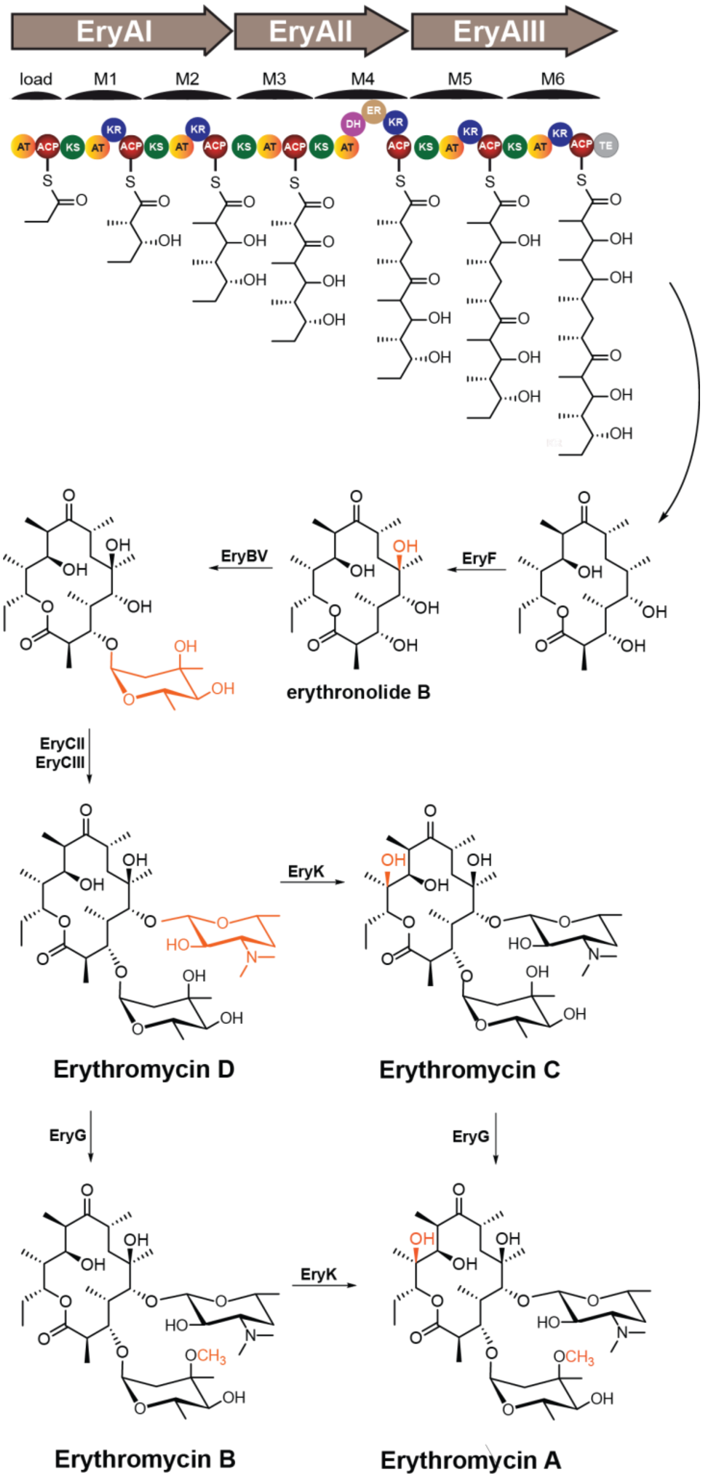

Figure S1. The erythromycin biosynthetic pathway (modified from [1–6]). The three enzymes EryAI, EryAII, and EryAIII are responsible for the initial loading of a propionyl-CoA starter unit and the subsequent elongation with six methylmalonyl-CoA extender units. The TE-catalysed cyclisation of the polyketide chain followed by the EryF-catalysed hydroxylation generate the precursor erythronolide. Next, the glycosyltransferases EryB and EryCII/CIIII catalyse the attachment of TDP-L-mycarose and TDP-D-desosamine, respectively, yielding erythromycin D. Finally, the conversion of erythromycin D to erythromycin A is orchestrated by EryG and EryK through the precursors erythromycin C or B. M, module; AT, acyltransferase; ACP, acyl carrier protein; KS, ketosynthase; KR, ketoreductase; DH, dehydratase; ER, enoyl reductase; TE, thioesterase; TDP, thymidine diphosphate.

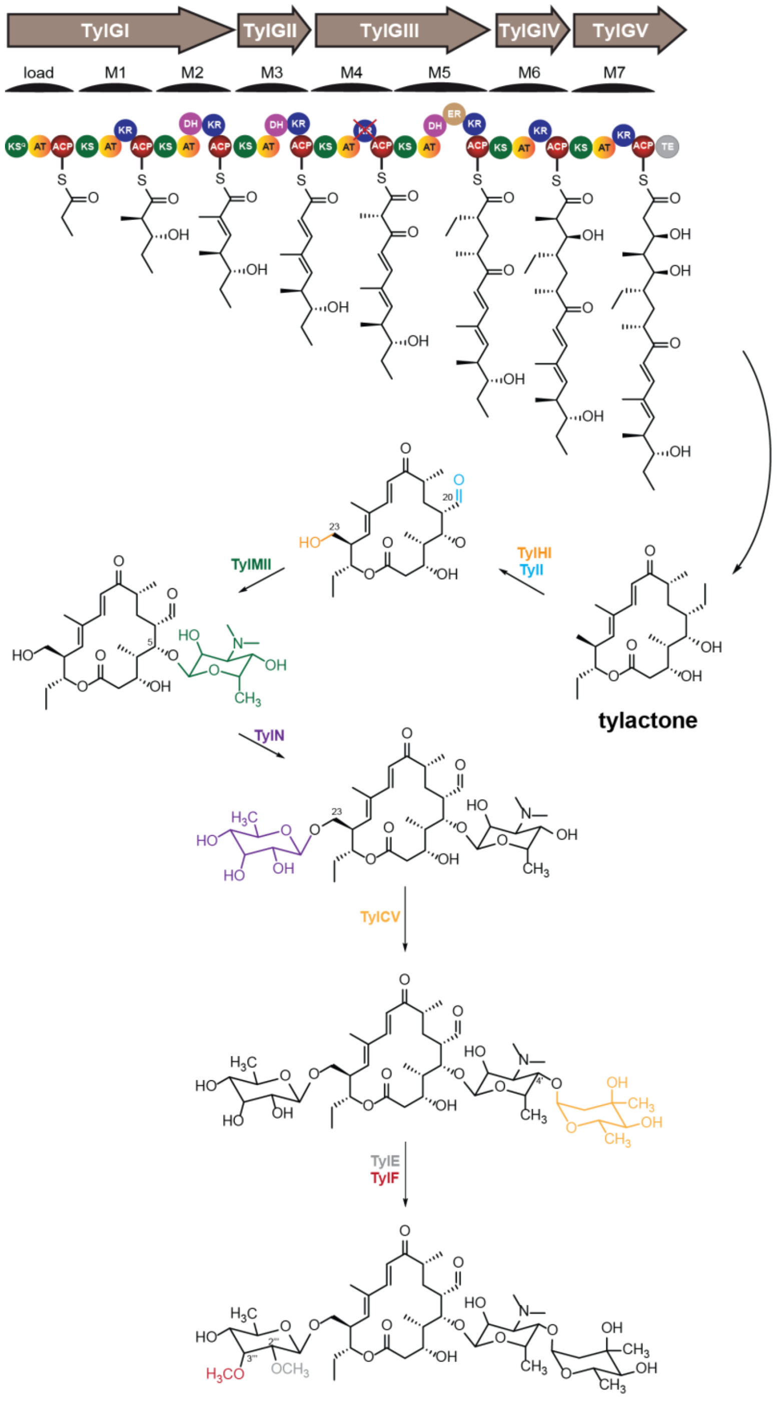

Tylosin A

Figure S2: The tylosin biosynthetic pathway (modified from [7–13]). The five enzymes TylGI through TylGV are responsible for the initial loading of a methylmalonyl-CoA which is extended by additional four methylmalonyl-CoAs, two malonyl-CoAs, and one ethylmalonyl-CoA. The TE-catalysed release of the aglycone leads to cyclisation resulting in the tylactone precursor. Additional post-modifications leading to tylosin A include hydroxylations by TylHI and TylI, the attachment of sugars d-mycosamine, mycinose, and L-mycarose by the glycosyltransferases TylMII, TylN, and TylCV, respectively, and finally, O-methylation of the mycinose by TylE and TylF. M, module; AT, acyltransferase; ACP, acyl carrier protein; KS, ketosynthase; KR, ketoreductase; DH, dehydratase; ER, enoyl reductase; TE, thioesterase.

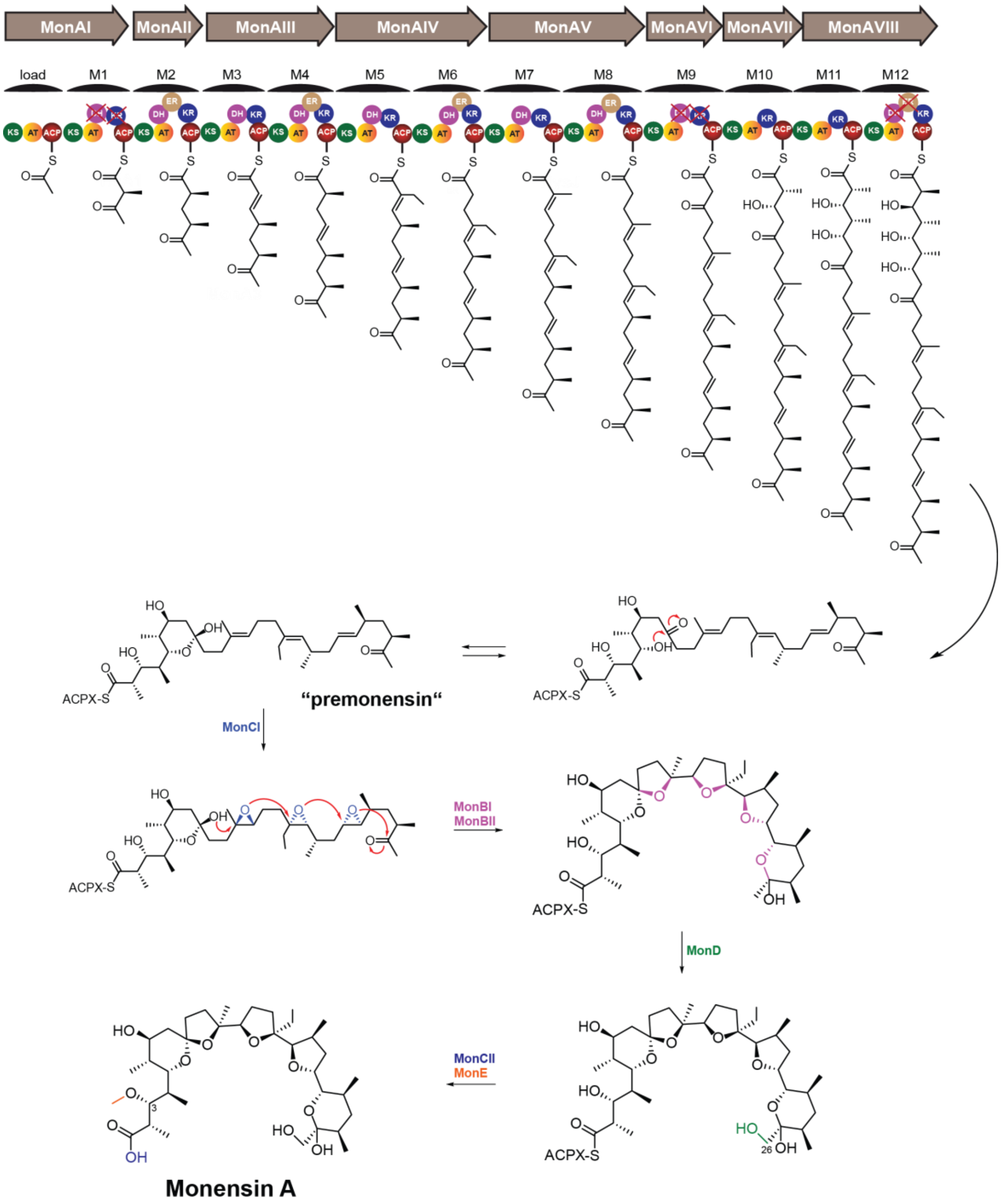

Figure S3: The monensin biosynthetic pathway (modified from [14,15]). The eight enzymes MonAI through MonAVIII orchestrate the initial loading of a malonyl-CoA starter unit followed by its elongation with four malonyl-CoA, one ethylmalonyl CoA, and seven propionyl-CoA extender units. The monensin gene cluster lacks the conventional thioesterase domain and instead the polyketide precursor is transferred to a discrete acyl carrier protein (ACPX), from which additional post modifications occur. These modifications include the MonCI-catalysed epoxidation, which is followed by epoxide hydroxylation mediated by MonBI and MonBII, and hydroxylation and O-methylation by MonD and MonE, respectively. Finally, the precursor is released from the ACPX by MonCII, yielding monensin A. M, module; AT, acyltransferase; ACP, acyl carrier protein; KS, ketosynthase; KR, ketoreductase; DH, dehydratase; ER, enoyl reductase; ACPX, discrete acyl carrier protein.

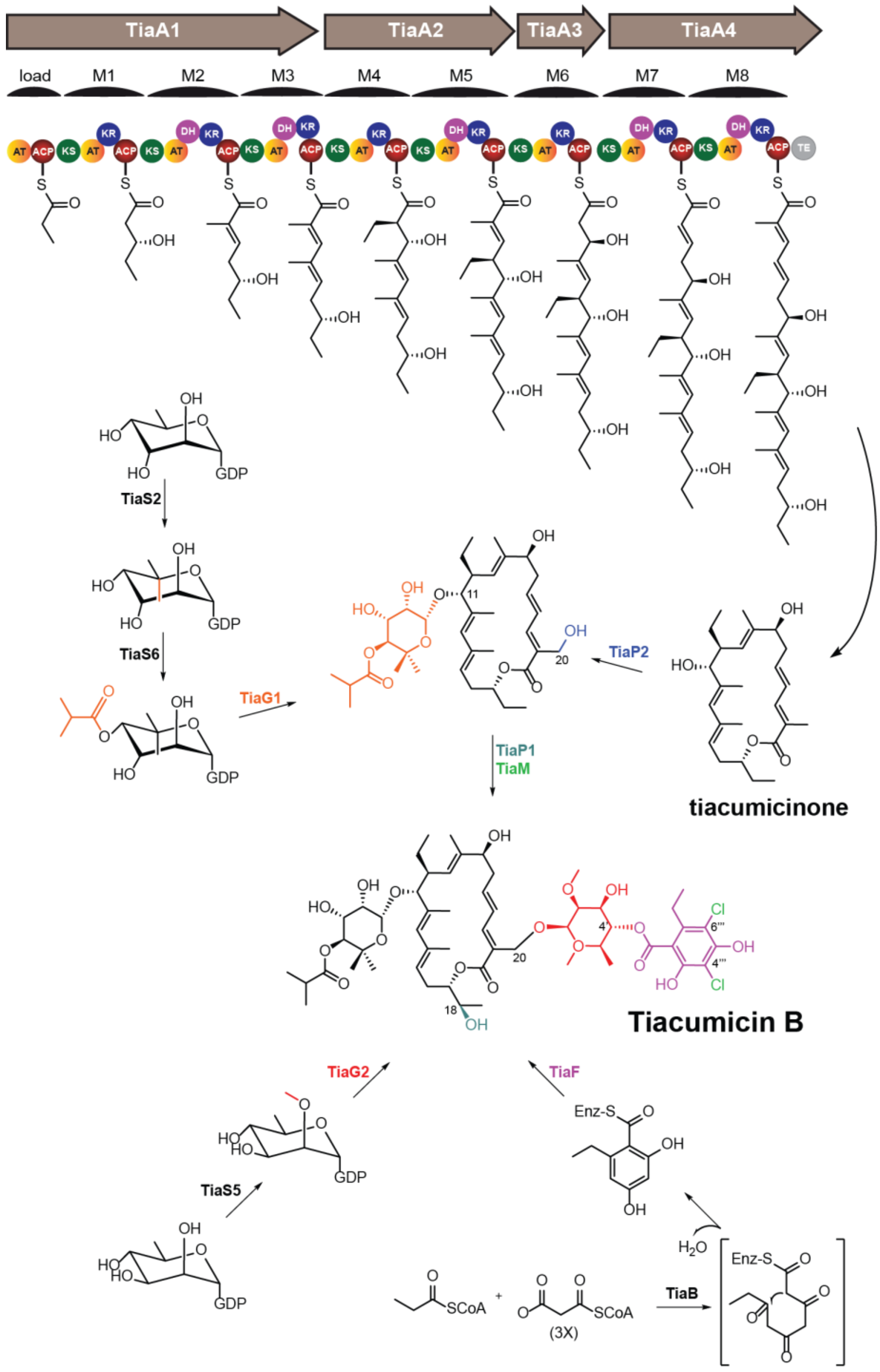

Figure S4: The biosynthetic pathway of tiacumicin (modified from [16,17]). The four enzymes TiaA1 through TiaA4 are responsible for the loading of a methylmalonyl-CoA starter unit followed by its elongation with three malonyl-CoAs, four methylmalonyl-CoAs, and one ethylmalonyl-CoA. The TE-facilitated release of the polyketide precursor leads to cyclisation and formation of tiacumicinone. Obtaining tiacumicin B requires the action of several modifying enzymes, including the two hydroxylases TiaP1 and TiaP2, the glycosyltransferases TiaG1 and TiaG2, responsible for installation of the two rhamnose sugars, and finally the acyltransferase TiaF and dihalogenase TiaM, which catalyse the transfer and subsequent dihalogenation of the homo-orsellinic acid moiety attached to the C20 rhamnose sugar. Additional enzymes responsible for sugar biosynthesis (TiaS2, TiaS6, TiaS5) and for synthesis of the homo-orsellinic acid (TiaB) are encoded by genes in the gene cluster of tiacumicin as well. M, module; AT, acyltransferase; ACP, acyl carrier protein; KS, ketosynthase; KR, ketoreductase; DH, dehydratase; TE, thioesterase; GDP, guanosine diphosphate.

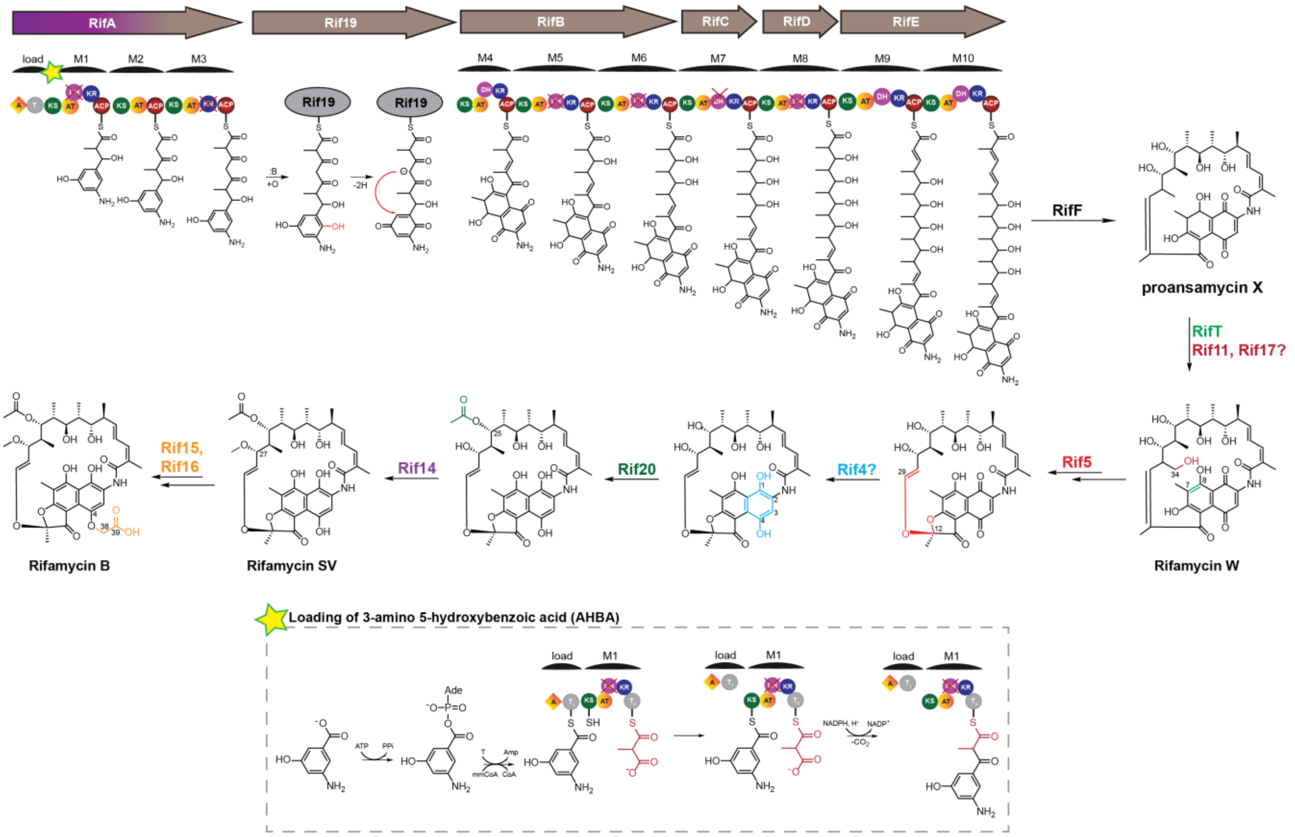

Figure S5: The biosynthetic pathway of rifamycin (modified from [18–26]). The product of the hybrid type I PKS/NRPS of rifamycin is proansamycin X. Assembly of this precursor starts with the loading of the unusual starter unit 3-amino 5-hydroxybenzoic acid (AHBA) onto RifA, a hybrid NRPS/PKS. Following the extension with one malonyl-CoA and one propionyl-CoA, the precursor is transferred to Rif19, which catalyse the ring closure leading to the naphthoquinone ring. The additional seven modules RifB through RifE further elongates the polyketide precursor with seven acetates and one propionate. The release of the polyketide precursor and formation of proansamycin X is facilitated by the amide synthase RifF. During post-modifications, proansamycin X is first converted to rifamycin W through dehydrogenation and hydroxylation catalysed by RifT and Rif11/Rif17, respectively. Next, the formation of the Δ12,29 olefinic bond is catalysed by the monooxygenase Rif5. Additional reduction of the quinone, acetylation of the hydroxyl group on C25, and O-methylation of C27 are orchestrated by Rif4, Rif20, and Rif14, respectively. Lastly, conversion of rifamycin SV to rifamycin B is facilitated by the enzymes Rif15 and Rif16, which install the glycolate at the C4 position. M, module; AT, acyltransferase; ACP, acyl carrier protein; KS, ketosynthase; KR, ketoreductase; DH, dehydratase.

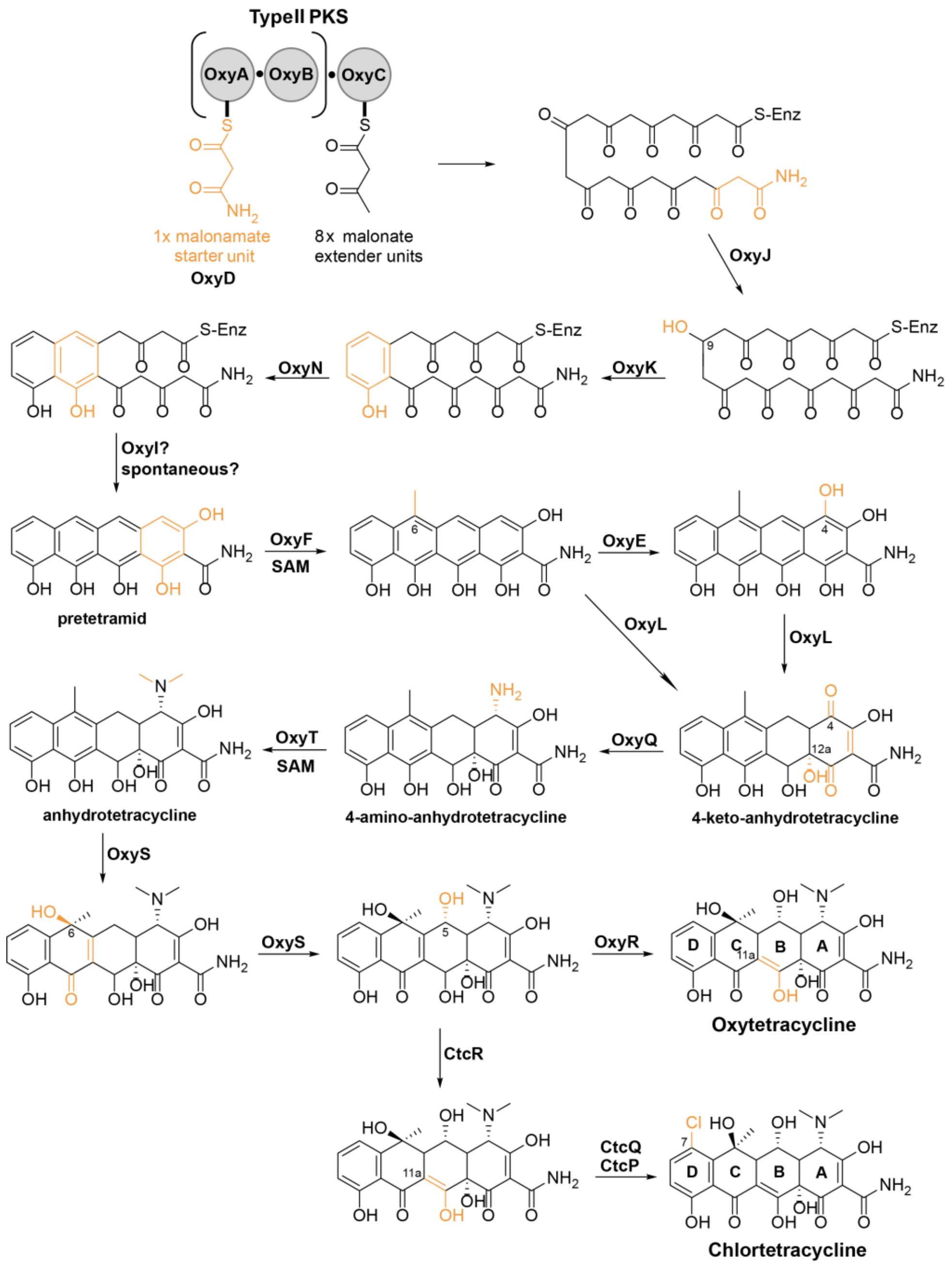

Figure S6: The biosynthetic pathway of oxytetracycline and chlortetracycline (modified from [27–33]). The biosynthesis of the structurally similar compounds is exemplified with biosynthesis of oxytetracycline which starts with the type II iterative PKS-facilitated (OxyA – OxyC) assembly of one malonamate starter unit with eight malonate extender units. Biosynthesis of the unusual starter unit is facilitated by OxyD. Upon its assembly, the polyketide precursor undergoes several modifications starting with cyclisation through ring closure, which in the case of the two first rings are facilitated by OxyK and OxyN and for the two last rings either is the result of spontaneous cyclisation or through the actions of the putative cyclase OxyI. The resulting pretetramid is methylated at C6 by OxyF and further dihydroxylated at positions C4/C12a by either OxyL alone or by the dual actions of OxyL and OxyE, leading to formation of 4-keto-anhydrotetracycline. The final steps leading to oxytetracycline includes the OxyQ-mediated transfer of an amino group to the C4, which subsequently undergoes dimethylation catalysed by OxyT. Finally, OxyS and OxyR catalyse the conversion of anhydrotetracycline to oxytetracycline through hydroxylation of ring C and B. In the case of chlortetracycline, the reduction of ring B is catalysed by CtcR, following which the final halogenation at C7 can occur by the dual actions of halogenase CtcP and the flavin reductase CtcP, responsible for provision of $FADH_2$ required for the reaction. PKS, polyketide synthase; SAM, S-Adenosyl methionine; $FADH_2$, dihydroflavine adenine dinucleotide.

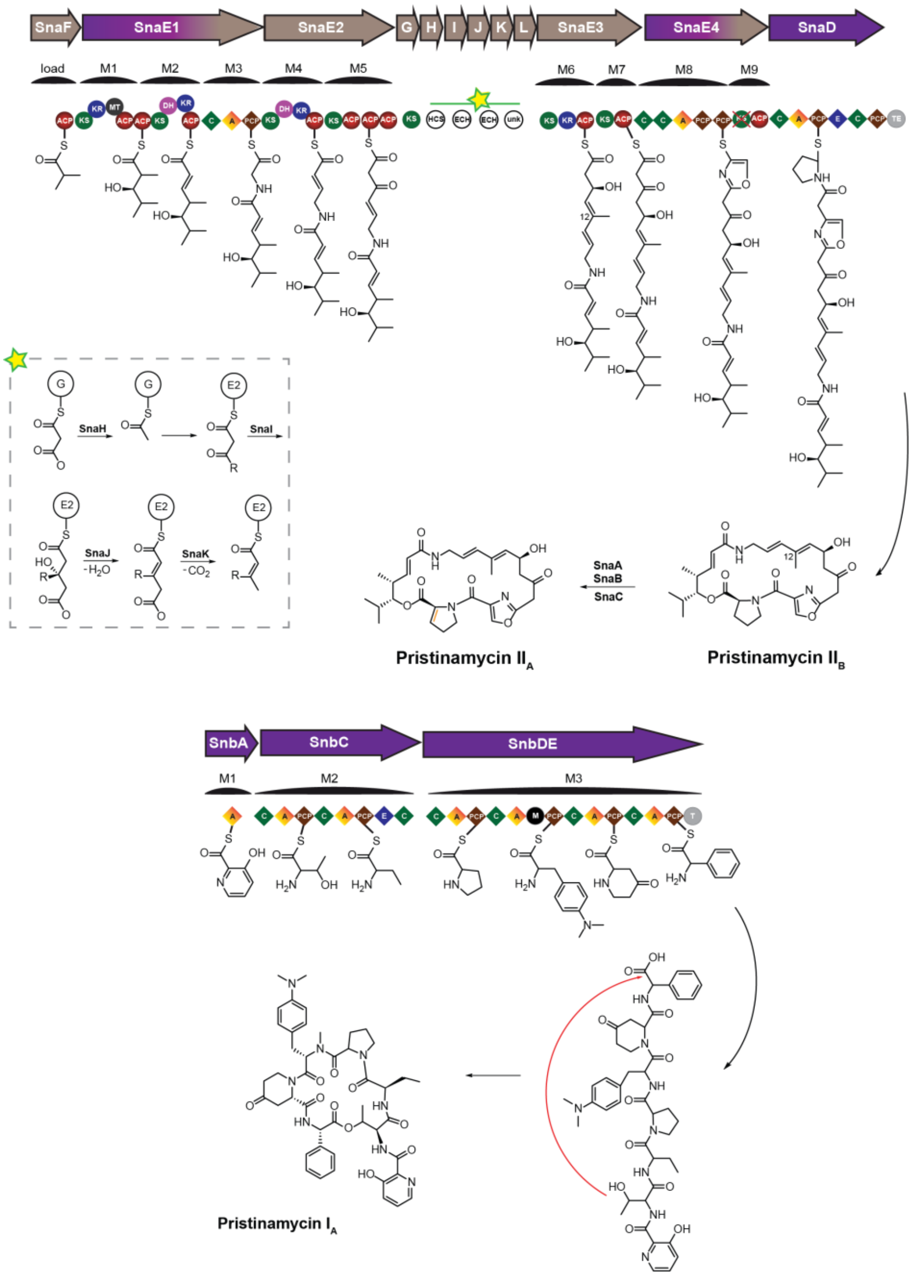

Figure S7: The biosynthetic pathways of pristinamycin II and pristinamycin I (modified from [34]). Biosynthesis of pristinamycin II is orchestrated by the hybrid NRPS/PKS composed of SnaF, SnaE1 through SnaE4, SnaD, and SnaF through SnaL. The assembly starts with loading of isobuturyl-CoA to SnaF and further elongation with two malonyl-CoA units and one glycine by the hybrid PKS/NRPS SnaE1 and two malonyl-CoA units by SnaE2. Next, SnaG through SnaL ensures the transfer of a methyl group to the C12 position in the growing chain, before further extension occurs, starting with addition of two acetate units by SnaE3, serine by hybrid NRPS/PKS SnaE4, and finally proline by the NRPS SnaD. The TE-mediated release and cyclisation of the NRPS/PKS chain generates pristinamycin IIB, which is converted into pristinamycin IIA by the monooxygenase subunits SnaA and SnaB together with the FMN reductase SnaC. Biosynthesis of pristinamycin I is orchestrated by the NRPSs SnbA, SnbC, and SnbDE. Following loading of starter unit 3-hydroxypicollinic acid, the peptide is elongated with L-threonine, L-aminobutyric acid, L-proline, 4-N,N-dimethylamino-L-phenylalanine, 4-oxo-l-pipecolic acid, and L-phenylglycine. Release and cyclisation of the peptide chain is facilitated by the TE in module 3, leading to formation of pristinamycin I. M, module; ACP, acyl carrier protein; KS, ketosynthase; KR, ketoreductase; MT, methyltransferase; DH, dehydratase; C, condensation; A, adenylation; PCP, peptidyl carrier protein; HCS, hydroxymethylglutaryl-CoA (HMG-CoA) synthase; ECH, enoyl-CoA hydratase; unk, unknown; TE, thioesterase; E, epimerase; T, thioesterase; FMN, flavin mono nucleotide.

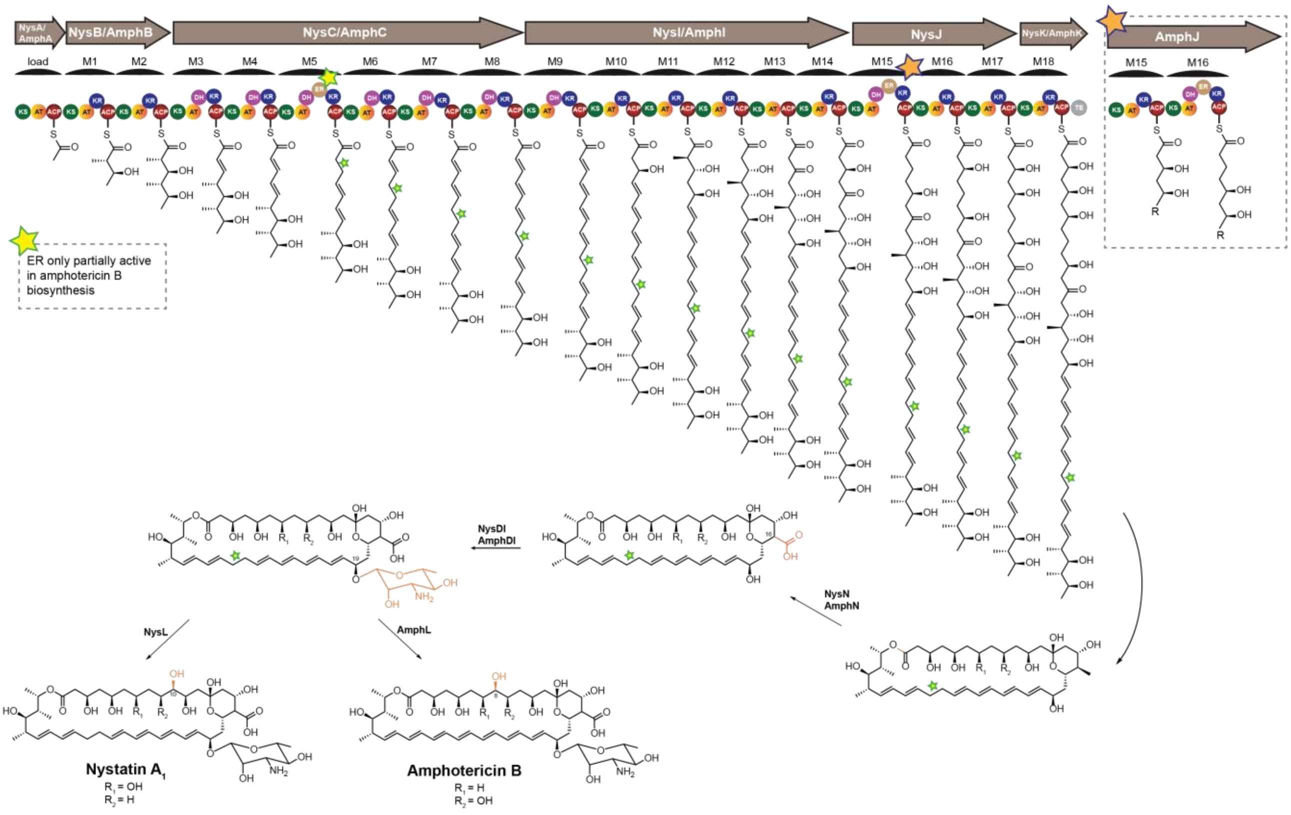

Figure S8: The biosynthetic pathways of nystatin and amphotericin B (modified from [35–40]). The two polyene share much of the same biosynthetic principle and only differ in the three modules M5, M15, and M16, as marked by coloured stars in the assembly lines. Overall, the PKS-mediated elongation of the polyenes is orchestrated by enzymes NysA through NysC and NysI through NysK in the case of nystatin and by AmphA through AmphC and AmphI through AmphK for amphotericin B biosynthesis. For both compounds, the elongation starts with loading of the starter unit acetyl-CoA to which three methylmalonyl-CoA and 15 malonyl-CoA extender units are subsequently attached. Following its TE-mediated release and cyclisation, the polyketide precursor undergoes oxidation of the methyl group on C16, glycosylation with a mycosamine on C19, and hydroxylation at position C10/C8, catalysed by NysN/AmphN, NysDI/AmphDI, and NysL/AmphL, respectively. M, module; AT, acyltransferase; ACP, acyl carrier protein; KS, ketosynthase; KR, ketoreductase; DH, dehydratase; ER, enoyl reductase; TE, thioesterase.

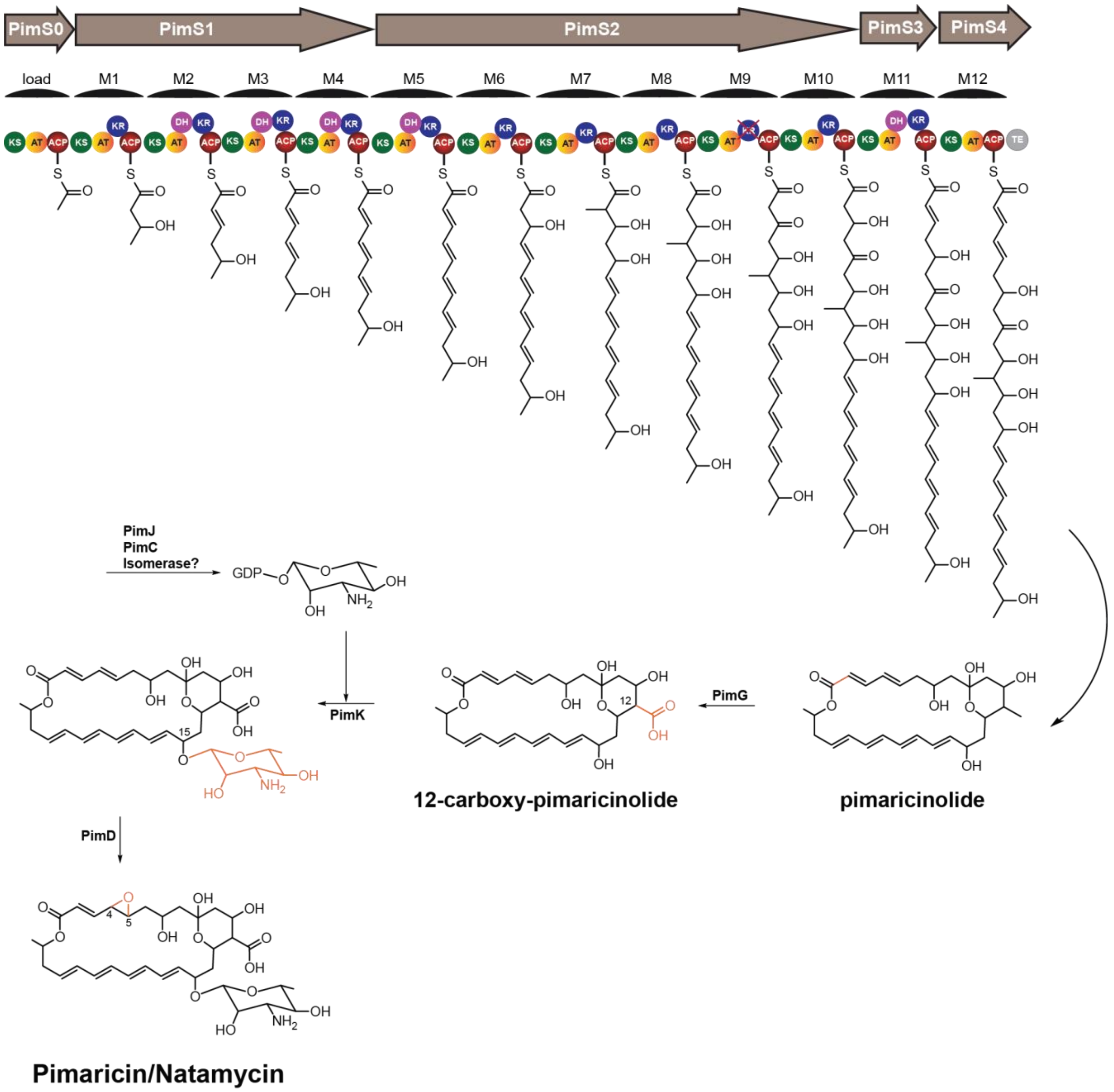

Figure S9: The biosynthetic pathway of pimaricin (modified from [41,42]). The five enzymes PimS0 through PimS4 are responsible for the loading of malonyl-CoA, which is further elongated by 12 malonyl-CoA extender units and one propionyl-CoA unit. Upon the TE-mediated release and cyclisation of the polyketide precursor, pimaricinolide is formed. This precursor is further modified by first PimG, responsible for the carboxylation leading to intermediate 12-carboxy-pimaricinolide, following which the glycosyl-transferase PimK transfers a mycosamine to the C15 position, and finally an epoxy group is spontaneously formed between C4 and C5 as a consequence of a hydroxylation by PimD. M, module; AT, acyltransferase; ACP, acyl carrier protein; KS, ketosynthase; KR, ketoreductase; DH, dehydratase; TE, thioesterase; GDP, guanosine diphosphate.

Supplementary References

1. Rawlings, B. J. Type I polyketide biosynthesis in bacteria (Part A—erythromycin biosynthesis). *Nat. Prod. Rep.* **2001,** *18*, 190–227.
2. Weissman, K. J. Genetic engineering of modular PKSs: from combinatorial biosynthesis to synthetic biology. *Nat. Prod. Rep.* **2016,** *33*, 203–230.
3. Cortes, J.; Haydock, S. F.; Roberts, G. A.; Bevitt, D. J.; Leadlay, P. F. An unusually large multifunctional polypeptide in the erythromycin-producing polyketide synthase of *Saccharopolyspora erythraea*. *Nature* **1990,** *348*, 176.
4. Donadio, S.; Staver, M.; McAlpine, J.; J Swanson, S.; Katz, L. Modular Organization of Genes Required for Complex Polyketide Biosynthesis. *Science* **1991,** *252*, 675–679.
5. Summers, R. G.; Donadio, S.; Staver, M. J.; Wendt-Pienkowski, E.; Hutchinson, C. R.; Katz, L. Sequencing and mutagenesis of genes from the erythromycin biosynthetic gene cluster of *Saccharopolyspora erythraea* that are involved in L-mycarose and D-desosamine production. *Microbiology* **1997,** *143*, 3251–3262.
6. Oliynyk, M.; Samborskyy, M.; Lester, J. B.; Mironenko, T.; Scott, N.; Dickens, S.; Haydock, S. F.; Leadlay, P. F. Complete genome sequence of the erythromycin-producing bacterium *Saccharopolyspora erythraea* NRRL23338. *Nat. Biotechnol.* **2007,** *25*, 447.
7. Lowell, A. N. *et al.* Chemoenzymatic Total Synthesis and Structural Diversification of Tylactone-Based Macrolide Antibiotics through Late-Stage Polyketide Assembly, Tailoring, and C-H Functionalization. *J. Am. Chem. Soc.* **2017,** *139*, 7913–7920.
8. Cundliffe, E.; Bate, N.; Butler, A.; Fish, S.; Gandecha, A.; Merson-Davies, L. The tylosin-biosynthetic genes of *Streptomyces fradiae*. *Antonie Van Leeuwenhoek* **2001,** *79*, 229–234.
9. Cox, K. L. *et al.* The use of recombinant DNA techniques to study tylosin biosynthesis and resistance in *Streptomyces fradiae*. *J. Nat. Prod.* **1986,** *49*, 971–980.
10. Fouces, R.; Mellado, E.; Diez, B.; Barredo, J. L. The tylosin biosynthetic cluster from *Streptomyces fradiae*: genetic organization of the left region. *Microbiology* **1999,** *145*, 855–868.
11. Gandecha, A. R.; Large, S. L.; Cundliffe, E. Analysis of four tylosin biosynthetic genes from the *tylLM* region of the *Streptomyces fradiae* genome. *Gene* **1997,** *184*, 197–203.
12. Merson-Davies, L. A.; Cundiiffe, E. Analysis of five tylosin biosynthetic genes from the *tyllBA* region of the *Streptomyces fradiae* genome. *Mol. Microbiol.* **1994,** *13*, 349–355.
13. Stratigopoulos, G.; Cundliffe, E. Expression Analysis of the Tylosin-Biosynthetic Gene Cluster: Pivotal Regulatory Role of the *tylQ* Product. *Chem. Biol.* **2002,** *9*, 71–78.
14. Hüttel, W.; Spencer, J. B.; Leadlay, P. F. Intermediates in monensin biosynthesis: a late step in biosynthesis of the polyether ionophore monensin is crucial for the integrity of cation binding. *Beilstein J. Org. Chem.* **2014,** *10*, 361–368.
15. Oliynyk, M. *et al.* Analysis of the biosynthetic gene cluster for the polyether antibiotic monensin in *Streptomyces cinnamonensis* and evidence for the role of *monB* and *monC* genes in oxidative cyclization. *Mol. Microbiol.* **2003,** *49*, 1179–1190.
16. Xiao, Y.; Li, S.; Niu, S.; Ma, L.; Zhang, G.; Zhang, H.; Zhang, G.; Ju, J.; Zhang, C. Characterization of tiacumicin B biosynthetic gene cluster affording diversified tiacumicin analogues and revealing a tailoring dihalogenase. *J. Am. Chem. Soc.* **2011,** *133*, 1092–1105.

17. Niu, S. *et al.* Characterization of a Sugar-O-methyltransferase TiaS5 Affords New Tiacumicin Analogues with Improved Antibacterial Properties and Reveals Substrate Promiscuity. *ChemBioChem* **2011,** *12*, 1740–1748.

18. Floss, H. G.; Yu, T.-W. Rifamycin mode of action, resistance, and biosynthesis. *Chem. Rev.* **2005,** *105*, 621–632.

19. Qi, F. *et al.* Deciphering the late steps of rifamycin biosynthesis. *Nat. Commun.* **2018,** *9*, 2342.

20. Kang, Q.; Shen, Y.; Bai, L. Biosynthesis of 3, 5-AHBA-derived natural products. *Nat. Prod. Rep.* **2012,** *29*, 243–263.

21. August, P. R. *et al.* Biosynthesis of the ansamycin antibiotic rifamycin: deductions from the molecular analysis of the rif biosynthetic gene cluster of *Amycolatopsis mediterranei* S699. *Chem. Biol.* **1998,** *5*, 69–79.

22. Admiraal, S. J.; Walsh, C. T.; Khosla, C. The Loading Module of Rifamycin Synthetase Is an Adenylation−Thiolation Didomain with Substrate Tolerance for Substituted Benzoates. *Biochemistry* **2001,** *40*, 6116–6123.

23. Yu, T.-W.; Shen, Y.; Doi-Katayama, Y.; Tang, L.; Park, C.; Moore, B. S.; Hutchinson, C. R.; Floss, H. G. Direct evidence that the rifamycin polyketide synthase assembles polyketide chains processively. *Proc. Natl. Acad. Sci.* **1999,** *96*, 9051–9056.

24. Yu, T.-W.; Müller, R.; Müller, M.; Zhang, X.; Draeger, G.; Kim, C.-G.; Leistner, E.; Floss, H. G. Mutational analysis and reconstituted expression of the biosynthetic genes involved in the formation of 3-amino-5-hydroxybenzoic acid, the starter unit of rifamycin biosynthesis in *Amycolatopsis mediterranei* S699. *J. Biol. Chem.* **2001,** *276*, 12546–12555.

25. Xu, J.; Wan, E.; Kim, C.-J.; Floss, H. G.; Mahmud, T. Identification of tailoring genes involved in the modification of the polyketide backbone of rifamycin B by *Amycolatopsis mediterranei* S699. *Microbiology* **2005,** *151*, 2515–2528.

26. White, R. J.; Martinelli, E.; Lancini, G. Ansamycin biogenesis: Studies on a novel rifamycin isolated from a mutant strain of *Nocardia mediterranei*. *Proc. Natl. Acad. Sci.* **1974,** *71*, 3260–3264.

27. Petković, H.; Lukežič, T.; Šušković, J. Biosynthesis of oxytetracycline by *Streptomyces rimosus*: past, present and future directions in the development of tetracycline antibiotics. *Food Technol. Biotechnol.* **2017,** *55*, 3–13.

28. Zhang, W.; Ames, B. D.; Tsai, S.-C.; Tang, Y. Engineered biosynthesis of a novel amidated polyketide, using the malonamyl-specific initiation module from the oxytetracycline polyketide synthase. *Appl. Environ. Microbiol.* **2006,** *72*, 2573–2580.

29. Thomas, R.; Williams, D. J. Oxytetracycline biosynthesis: origin of the carboxamide substituent. *J. Chem. Soc. Chem. Commun.* **1983,** 677–679.

30. Wang, P.; Gao, X.; Chooi, Y.-H.; Deng, Z.; Tang, Y. Genetic characterization of enzymes involved in the priming steps of oxytetracycline biosynthesis in *Streptomyces rimosus*. *Microbiology* **2011,** *157*, 2401–2409.

31. Pickens, L. B.; Tang, Y. Oxytetracycline biosynthesis. *J. Biol. Chem.* **2010,** *285*, 27509–27515.

32. Wang, P.; Bashiri, G.; Gao, X.; Sawaya, M. R.; Tang, Y. Uncovering the enzymes that catalyze the final steps in oxytetracycline biosynthesis. *J. Am. Chem. Soc.* **2013,** *135*, 7138–7141.

33. Zhu, T.; Cheng, X.; Liu, Y.; Deng, Z.; You, D. Deciphering and engineering of the final step halogenase for improved chlortetracycline biosynthesis in industrial *Streptomyces aureofaciens*. *Metab.*

Eng. **2013,** *19*, 69–78.

34. Mast, Y.; Weber, T.; Gölz, M.; Ort-Winklbauer, R.; Gondran, A.; Wohlleben, W.; Schinko, E. Characterization of the 'pristinamycin supercluster'of *Streptomyces pristinaespiralis*. *Microb. Biotechnol.* **2011,** *4*, 192–206.

35. Fjærvik, E.; Zotchev, S. B. Biosynthesis of the polyene macrolide antibiotic nystatin in *Streptomyces noursei*. *Appl. Microbiol. Biotechnol.* **2005,** *67*, 436–443.

36. Caffrey, P.; Lynch, S.; Flood, E.; Finnan, S.; Oliynyk, M. Amphotericin biosynthesis in *Streptomyces nodosus*: deductions from analysis of polyketide synthase and late genes. *Chem. Biol.* **2001,** *8*, 713–723.

37. Brautaset, T.; Sekurova, O. N.; Sletta, H.; Ellingsen, T. E.; Strøm, A. R.; Valla, S.; Zotchev, S. B. Biosynthesis of the polyene antifungal antibiotic nystatin in *Streptomyces noursei* ATCC 11455: analysis of the gene cluster and deduction of the biosynthetic pathway. *Chem. Biol.* **2000,** *7*, 395–403.

38. McNamara, C.; Crawforth, J.; Hickman, B.; Norwood, T.; Rawlings, B. Biosynthesis of amphotericin B. *J. Chem. Soc. Perkin Trans. 1* **1998,** 83–88.

39. Carmody, M.; Byrne, B.; Murphy, B.; Breen, C.; Lynch, S.; Flood, E.; Finnan, S.; Caffrey, P. Analysis and manipulation of amphotericin biosynthetic genes by means of modified phage KC515 transduction techniques. *Gene* **2004,** *343*, 107–115.

40. Carmody, M.; Murphy, B.; Byrne, B.; Power, P.; Rai, D.; Rawlings, B.; Caffrey, P. Biosynthesis of amphotericin derivatives lacking exocyclic carboxyl groups. *J. Biol. Chem.* **2005,** *280*, 34420–34426.

41. Aparicio, J. F.; Barreales, E. G.; Payero, T. D.; Vicente, C. M.; de Pedro, A.; Santos-Aberturas, J. Biotechnological production and application of the antibiotic pimaricin: biosynthesis and its regulation. *Appl. Microbiol. Biotechnol.* **2016,** *100*, 61–78.

42. Aparicio, J. F.; Fouces, R.; Mendes, M. V; Olivera, N.; Martı́n, J. F. A complex multienzyme system encoded by five polyketide synthase genes is involved in the biosynthesis of the 26-membered polyene macrolide pimaricin in *Streptomyces natalensis*. *Chem. Biol.* **2000,** *7*, 895–905.
